# Supplementary material for: Separation of Dihydro-Isocoumarins and Dihydro-Stilbenoids from Hydrangea macrophylla ssp. serrata by Use of Counter-Current Chromatography
Source: Molecules. 2022 May 26;27(11):3424. doi: 10.3390/molecules27113424 (PMC9182509; doi:10.3390/molecules27113424)
Supplement: Supplementary file 1 [file molecules-27-03424-s001.zip › supplementalfile_S1_dihydroisocoumarins.pdf]

Supplemental File S1: NMR and mass spectrometry of dihydroisocoumarins

Table A1: NMR assignments of hydrangenol 8-O-glc (1), thunberginol G-3'-glc (2) and phyllodulcin 8-O-glc (3)

	**hydrangenol 8-O-glc (1)**			**thunberginol G-3'-glc (2)**			**phyllodulcin 8-O-glc (3)**		
**No**	**δ_H (Multiplicity, *J*, nH) ¥**	**δ_C ¥**	**HMBC**	**δ_H (Multiplicity, *J*, nH) ¥**	**δ_C ¥**	**HMBC**	**δ_H (Multiplicity, *J*, nH)**	**δ_C**	**HMBC**
1	-	162.8/161.2	-	-	169.1/ 169.2	-	-	162.7	-
3	5.48/5.40 (dd, 11.6,2.6 Hz, 1H)	79.0/78.4	141.7/142.0(10), 128.2(6',2')	5.62/5.64 (dd, 12.4,3.2 Hz, 1H)	80.2/ 80.4	121.5/121.7(6'), 129.0/129.0(1'), 140.5/140.5(10)	5.45 (dd, 12.3,3.2 Hz, 1H)	78.8	113.9(2'), 117.5(6'), 130.8(1'), 141.9(10)
4a	3.35/3.35 (m, 1H)	34.7/34.8	-	3.42/ 3.42 (m, 1H)	33.2/ 33.2	80.2/80.4(3), 140.5/140.5(10)	3.3 (m, 1H)	34.9	-
4b	3.09/3.08 (dd, 16.4,2.6 Hz, 1H)	34.7/34.8	114.1/114.4(9), 120.4/121.6(5), 141.7/142.0(10)	3.16/ 3.16 (m, 1H)	33.2/ 33.2	108.3/108.4(9), 118.3/118.3(5), 140.5/140.5(10)	3.11 (dd, 16.7,2.6 Hz, 1H)	34.9	114.4(9),121.6(5), 141.9(10)
5	7.11/7.02 (d, 7.5 Hz, 1H)	121.7/120.5	34.7/34.8(4), 114.1/114.4(9), 116.5(7)	6.87/ 6.87 (m, 1H)	118.3/ 118.3	33.2/33.2(4), 108.3/108.4(9)	7.11 (d, 7.6 Hz, 1H)	121.6	34.9(4), 114.4(9),116.5(7)
6	7.60/7.53 (dd, 8.5,7.5 Hz, 1H)	134.9/134.3	141.7/142.0(10), 158.2/158.8(8)	7.53/ 7.53 (dd, 8.4,7.4 Hz, 1H)	136.2/ 136.2	140.5/140.5(10), 160.8/160.8(8)	7.60 (dd, 8.5,7.6 Hz, 1H)	134.9	141.9(10), 158.8(8)
7	7.33/7.19 (d, 8.5 Hz, 1H)	116.5/114.4	120.5/121.7(5)	6.90 /6.90 (d, 8.4 Hz, 1H)	115.3/ 115.3	118.3/118.3(5), 160.8/160.8(8)	7.33 (d, 8.5 Hz, 1H)	116.5	114.4(9),121.6(5), 158.8(8)
8	-	158.8/158.2	-	-	160.8/ 160.8	-	-	158.8	-
9	-	114.4/114.1	-	-	108.3/ 108.4	-	-	114.4	-
10	-	142.0/141.7	-	-	140.5/ 140.5	-	-	141.9	-
11	-	-	-	-	-	-	3.78 (s, 3H)	55.6	147.8(4')
1'	-	128.6/128.8	-	-	129.0/ 129.0	-	-	130.8	-
2'	7.33/7.31 (m, 1H)	128.2/128.0	78.4/79.0(3), 157.5/157.6(4')	7.29/ 7.29 (m/ 1H)	115.2/ 115.3	80.2/80.4(3), 121.5/121.7(6'), 145.0/145.1(3'), 147.1/147.2(4')	6.94 (d, 2.1 Hz, 1H)	113.9	117.5(6')
3'	6.81/6.78 (m, 1H)	115.0/115.0	128.6/128.8(1'), 157.5/157.6(4')	-	145.0/ 145.1	-	-	146.3	-
4'	-	157.6/157.5	-	-	147.1/ 147.2	-	-	147.8	-
5'	6.81/6.78 (m, 1H)	115.0/115.0	128.6/128.8(1'), 157.5/157.6(4')	6.86/ 6.86 (m, 1H)	115.6/ 115.7	129.0/129.0(1'), 145.0/145.1(3')	6.95 (d, 8.1 Hz, 1H)	111.8	130.8(1'), 146.3(3')
6'	7.33/7.31 (m, 1H)	128.2/128.0	78.4/79.0(3), 157.5/157.6(4')	7.04/ 7.05 (m, 1H)	121.5/ 121.7	80.2/80.4(3), 147.1/147.2(4')	6.90 (dd, 8.1,2.1 Hz, 1H)	117.5	78.8(3), 113.9(2'),147.8(4')
A1	4.86/4.95 (d, 7.6 Hz, 1H)	103.3/100.3	158.2/158.8(8)	4.70/ 4.71 (d, 7.5 Hz, 1H)	101.9/ 102.3	145.0/145.1(3')	4.86 (d, 7.2 Hz, 1H)	103.2	158.8(8)
A2	3.35/3.35 (m, 1H)	73.1/73.5	-	3.3/ 3.3 (m, 1H)	73.2/ 73.2	-	3.3 (m, 1H)	73.5	-
A3	3.35/3.27 (m, 1H)	75.6/76.5	-	3.3/ 3.3 (m, 1H)	75.9/ 75.9	-	3.3 (m, 1H)	75.6	-
A4	3.19/3.19 (m, 1H)	69.6/69.4	75.6/76.5(3)	3.16/ 3.16 (m/ 1H)	69.7/ 69.8	-	3.18 (m, 1H)	69.6	-
A5	3.35/3.35 (m, 1H)	77.5/77.0	-	3.3/ 3.3 (m, 1H)	77.0/ 77.2	-	3.39 (m, 1H)	77.5	60.7(A6), 103.2(A1)
A6a	3.75/3.71 (m, 1H)	60.7/60.6	69.4/69.6(A4)	3.71 (m, 1H)	60.5/ 60.6	-	3.74 (m, 1H)	60.7	-
A6b	3.49/3.49 (m, 1H)	60.7/60.6	-	3.48 (m, 1H)	60.5/ 60.6	-	3.51 (m, 1H)	60.7	77.5(A5)

¥ Compounds were obtained as position 3 diastereomers. Signals could not be unequivocally assigned.

Table A2: NMR assignments of hydrangenol-4'-O-glc (4) and phyllodulcin 3'-O-glc (5)

	**hydrangenol-4'-O-glc (4)**			**phyllodulcin 3'-O-glc (5)**		
**No**	**δ H (Multiplicity, *J*, nH) ¥**	**δ C¥**	**HMBC**	**δ H (Multiplicity, *J*, nH) ¥**	**δ C¥**	**HMBC**
1	-	169.0/169.0	-	-	169.1/169.1	-
3	5.71/ 5.71 (dd, 12.1,3.2 Hz, 1H)	79.8/ 79.9	128.0(2'), 128.0(6')	5.66/5.67 (dd, 11.8,3.2 Hz, 1H)	80.0/80.0	-
4a	3.38/ 3.38 (m, 1H)	33.8/ 33.8	140.5(10)	3.41/3.41 (m, 1H)	33.6/33.5	80.0(3), 108.5(9), 117.9(5), 130.6(1'), 140.5(10)
4b	3.15/ 3.15 (m, 1H)	33.8/ 33.8	108.6(9),140.5(10)	3.15/3.15 (m, 1H)	33.6/33.5	108.5(9), 117.9(5), 140.5(10)
5	6.80/ 6.80 (d, 7.4 Hz, 1H)	117.6/ 117.6	33.8(4), 108.6(9)	6.83/6.83 (m, 1H)	117.9/117.9	33.5/33.6(4), 108.5(9), 115.8(7)
6	7.49/ 7.49 (dd, 8.4,7.3 Hz, 1H)	136.2/ 136.2	140.5(10), 161.9(8)	7.51/7.51 (6.8,7.3 Hz, 1H)	136.3/136.3	140.5(10), 161.5(8)
7	6.86/ 6.86 (d, 8.4 Hz, 1H)	116.2/ 116.2	108.6(9),117.6(5)	6.87/6.87 (d, 8.3 Hz, 1H)	115.8/115.8	108.5(9),117.9(5)
8	-	161.9/ 161.9	-	-	161.5/161.5	-
9	-	108.6/ 108.6	-	-	108.5/108.5	-
10	-	140.5/ 140.5	-	-	140.5/140.5	-
11	-	131.7/ 131.7	-	3.8/3.78 (s, 3H)	55.7/55.7	149.2(4')
1'	7.45/ 7,45 (m, 1H)	128.0/ 128.0	-	-	130.6/130.6	-
2'	7.08/ 7.08 (m, 1H)	116.2/ 116.2	79.8/79.9(3), 157.6(4')	7.23/7.23 (m, 1H)	114.2/114.2	80.0(3),120.2/120.6(6'), 146.3(3'), 149.2(4')
3'	-	157.6/ 157.6	131.7(1'),157.6(4')	-	146.3/146.3	-
4'	7.08 (m, 1H)	116.2, 116.2	-	-	149.2/149.2	-
5'	7.45	128.0, 128.0	131.7(1'),157.6(4')	7.03/7.03 (d, 8.4 Hz, 1H)	112.2/112.3	130.6(1'),146.3(3')
6'	4.90/ 4.90 (d, 7.5 Hz, 1H)	100.2/ 100.2	79.8/79.9(3), 157.6(4')	7.09/7.09 (m, 1H)	120.6/120.2	80.0(3), 114.2(2'),149.2(4')
A1	3.24/ 3.24 (m, 1H)	73.2/ 73.2	157.6(4')	4.92/4.92 (d, 7.8 Hz, 1H)	100.1/100.1	146.3(3')
A2	3.28/ 3.28 (m, 1H)	76.6/ 76.6	76.6(A3), 100.2(A1)	3.31/3.31 (m, 1H)	73.2/73.2	-
A3	3.15/ 3.15 (m, 1H)	69.7/ 69.7	69.7(A4), 73.2(A2)	3.31/3.31 (m, 1H)	77.0/77.0	-
A4	3.33/ 3.33 (m, 1H)	77.1/ 77.1	-	3.15/3.15 (m, 1H)	69.6/69.6	-
A5	3.69/ 3.69 (m, 1H)	60.7/ 60.7	-	3.31/3.31 (m, 1H)	77.1/77.1	-
A6a	3.46/ 3.46 (m, 1H)	60.7/ 60.7	-	3.67/3.67 (m, 1H)	60.5/60.5	69.6(A4)
A6b	-	169.0/169.0	77.1(A5)	3.46/3.46 (m, 1H)	60.5/60.5	

¥ Compounds were obtained as position 3 diastereomers. Signals could not be unequivocally assigned.

Table A3: NMR assignments of thunberginol C-8-O-glc (6), thunberginol E-6-O-glc (7) and thunberginol D-6-O-glc (8)

	**thunberginol C-8-O-glc (6)**			**thunberginol E-6-O-glc (7)**			**thunberginol D-6-O-glc (8)**		
**No**	**δ_H (Multiplicity, *J*, nH) ¥**	**δ_C ¥**	**HMBC**	**δ_H (Multiplicity, *J*, nH)**	**δ_C**	**HMBC**	**δ_H (Multiplicity, *J*, nH) ¥**	**δ_C ¥**	**HMBC**
1	-	161.6/161.6	-	-	169.2	-	-	169.1/169.2	-
3	5.38/5.33 (dd, 11.2,2.9 Hz, 1H)	78.6/77.8	-	5.60 (dd, 11.4,3.3 Hz, 1H)	79.7	113.9(2'), 117.6(6'), 130.8(1'), 142.1(10)	5.53/5.55 (dd, 11.9, 3.3 Hz, 1H)	79.9/79.9	114.1(2'), 117.7(6'), 128.9/129.0(1'), 142.0(10)
4a	3.21/3.21 (m, 1H)	35.2/35.1	-	3.29 (m, 1H)	33.8	-	3.34/ 3.34 (m, 1H)	33.7/33.7	-
4b	2.99/3.00 (m, 1H)	35.2/35.1	105.8/105.9(9), 107.9/108.9(5) 143.5/143.9(10)	3.12 (dd, 16.8,3.3 Hz, 1H)	33.8	102.6(9), 107.3(5), 142.1(10)	3.09/ 3.09 (m, 1H)	33.7/33.7	102.5(9), 107.2/107.3(5),142.0(10)
5	6.49/6.41 (m, 1H)	108.9/107.9	35.1/35.2(4), 102.3(7), 105.8/105.9(9)	6.55 (d, 2.3 Hz, 1H)	107.3	33.8(4), 102.6(9)	6.55/6.55 (m,1H)	107.2/ 107.3	33.7(4), 102.5/ 102.5(9)
6	-	163.3/162.8	-	-	163.3	-	-	162.9/162.9	-
7	6.72/6.57 (m, 1H)	104.1/102.3	105.8/105.8(9), 107.9/108.9(5), 160.7/161.5(8),162.8//163.3(6)	6.51 (d, 2.3 Hz, 1H)	101.8	102.6(9), 107.3(5),163.3(8)	6.51/ 6.53 (m, 1H)	101.6/101.6	102.5/ 102.5(9), 107.2/ 107.3(5)
8	-	161.5/160.7	-	-	163.0	-	-	163.2/163.2	-
9	-	105.9/105.8	-	-	102.6	-	-	102.5/ 102.5	-
10	-	143.9/143.5	-	-	142.1	-	-	142.0/142.0	-
11	-	-	-	3.79 (s, 3H)	55.7	147.9(4')	-	-	-
1'	-	128.9/129.2	-	-	130.8	-	-	128.9/ 129.0	-
2'	7.31/7.27 (m, 1H)	128.2/128.0	77.8/78.6(3), 157.5(157.6(4')	6.92 (d, 2.1 Hz, 1H)	113.9	79.7(3), 117.6(6'), 147.9(4')	6.88/6.88 (m, 1H)	114.1/114.1	79.9(3), 117.7(6'), 145.6(4')
3'	6.80/6.77 (m, 1H)	115.1/115.1	128.9/129.2(1'), 157.5/157.6(4')	-	146.5	-	-	145.1/145.1	79.9(3), 117.7(6'), 145.6(4')
4'	-	157.6/157.5	-	-	147.9	-	-	145.6/145.6	-
5'	6.80/6.77 (m, 1H)	115.1/115.1	128.9/129.2(1'), 157.5/157.6(4')	6.95 (d, 8.4 Hz, 1H)	112	130.8(1'), 146.5(3')	6.75/6.75 (m, 1H)	115.3/115.3	-
6'	7.31/7.27 (m, 1H)	128.2/128.0	77.8/78.6(3), 157.5(157.6(4')	6.88 (dd, 8.4,2.1 Hz, 1H)	117.6	79.7(3), 113.9(2'), 147.9(4')	6.75/6.75 (m, 1H)	117.7/117.7	-
A1	4.74/4.82 (d, 7.4 Hz, 1H)	103.9/101.0	160.7/161.5(8)	4.98 (d, 7.5 Hz, 1H)	99.8	163.3(6)	4.97/4.99 (d, 7.8 Hz, 1H)	99.6/ 99.5	163.2 (6)
A2	3.34/3.34 (m, 1H)	73.7/73.2	-	3.23 (m, 1H)	73.1	99.8(A1)	3.23/3.23 (m, 1H)	73.0/73.0	76.4(A3), 99.5/99.6(A1)
A3	3.34/3.34 (m, 1H)	75.5/76.5	-	3.29 (m, 1H)	76.5	-	3.34/3.34 (m, 1H)	76.4/76.4	-
A4	3.21/3.21 (m, 1H)	69.6/69.4	-	3.17 (m, 1H)	69.5	-	3.17/3.17 (m, 1H)	69.4/69.4	60.4(A6), 76.4(A3)
A5	3.34/3.34 (m, 1H)	77.6/76.5	-	3.39 (m, 1H)	77.2	-	3.34/3.34 (m, 1H)	77.0/ 77.0	-
A6a	3.75/3.71 (m, 1H)	60.7/60.6	-	3.7 (m, 1H)	60.5	-	3.68/3.69 (m, 1H)	60.4/60.4	69.4(A4),
A6b	3.54/3.54 (m, 1H)	60.7/60.6	-	3.46 (m, 1H)	60.5	77.2(A5)	3.45/3.45 (m, 1H)	60.4/60.4	77.0(A5)

¥ Compounds were obtained as position 3 diastereomers. Signals could not be unequivocally assigned.

Table A4: NMR assignments of thunberginol C (9), thunberginol E (10) and thunberginol G (11)

	**thunberginol C (9)**			**thunberginol E (10)**			**thunberginol G (11)**		
**No**	**δ H (Multiplicity, *J*, nH)**	**δ C**	**HMBC**	**δ H (Multiplicity, *J*, nH)**	**δ C**	**HMBC**	**δ H (Multiplicity, *J*, nH)**	**δ C**	**HMBC**
1	-	169.6	-	-	169.4	-	-	169.4	-
3	5.54 (dd, 12.1,3.2 Hz, 1H)	79.9	128.3(2'), 128.3(6'),142.5(10)	5.52 (dd, 11.7,3.3 Hz, 1H)	79.5	113.9(2'), 117.5(6'), 131.1(1'), 142.1(10)	5.53 (dd, 12.0,3.2 Hz, 1H)	80.5	114.3(2'),117.9(6'),129.1(1')
4a	3.27 (dd, 16.6,12.2 Hz, 1H)	33.8	79.9(3), 100.4(9),107.0(5), 142.5(10)	3.02 (dd, 16.8,3.3Hz, 1H)	33.8	79.9(3), 142.1(10)	3.26 (m, 1H)	33.6	80.5(3),140.6(10)
4b	3.02 (dd, 16.6,3.1 Hz, 1H)	33.8	100.4(9),107.0(5), 142.5(10)	3.2 (dd, 16.8,11.7 Hz, 1H)	33.8	99.7(9), 107.3(5), 142.1(10)	3.06 (dd, 16.6,3.2 Hz, 1H)	33.6	108.5(9),118.4(5),140.6(10)
5	6.30 (d, 1.8 Hz, 1H)	107.0	33.8(4), 100.4(9), 101.0(7), 164.6(6)	6.27 (d, 2.2 Hz, 1H)	107.3	33.8(4), 99.7(9), 101.1(7)	6.80 (d, 7.4 Hz, 1H)	118.4	33.6(4),108.5(9),136.3(6)
6	-	164.6	-	-	165.7	-	7.45 (dd, 8.4,7.4 Hz, 1H)	136.3	140.6(10),160.9(8)
7	6.24 (d, 2.2 Hz, 1H)	101.0	100.4(9), 107.0(5)	6.19 (d, 2.2 Hz, 1H)	101.1	107.3(5), 163.5(8),165.7(6)	6.82 (d, 8.4 Hz, 1H)	115.4	118.4(5)
8	-	163.5	-	-	163.5	-	-	160.9	-
9	-	100.4	-	-	99.7	-	-	108.5	-
10	-	142.5	-	-	142.1	-	-	140.6	-
11	-	-	-	3.78 (s, 3H)	55.6	147.9(4')	-	-	-
1'	-	128.7	-	-	131.1	-	-	129.1	-
2'	7.31 (m, 1H)	128.3	79.9(3), 157.8(4')	6.92 (d, 2.1 Hz, 1H)	113.9	117.5(6'), 147.9(4')	6.82 (m, 1H)	114.3	80.5(3)
3'	6.80 (m, 1H)	115.2	128.7(1')	-	146.5	-	-	145.2	-
4'	-	157.8	-	-	147.9	-	-	145.8	-
5'	6.80 (m, 1H)	115.2	128.7(1')	6.94 (d, 8.3 Hz, 1H)	112.0	131.1(1'), 146.5(3')	6.69 (m, 1H)	115.4	
6'	7.31 (m, 1H)	128.3	79.9(3), 157.8(4')	6.87 (dd, 8.3,2.1 Hz, 1H)	117.5	79.5(3), 113.9(2'), 147.9(4')	6.69 (m, 1H)	117.9	

Table A5: NMR assignments of hydrangenol (12) and phyllodulcin (13)

	hydrangenol (12)			phyllodulcin (13)		
**No**	**δ H (Multiplicity, *J*, nH)**	**δ C**	**HMBC**	**δ H (Multiplicity, *J*, nH)**	**δ C**	**HMBC**
1	-	169.3	-	-	169.3	-
3	5.66 (dd, 12.4,3.1 Hz, 1H)	80.4	128.2(2'), 128.2(6'), 140.5(10)-	5.65 (dd, 11.9,3.2 Hz, 1H)	80.2	114.0(2'), 117.6(6'), 130.8(1'), 140.5(10)
4a	3.40 (dd, 16.6,12.4 Hz, 1H)	33.4	80.4(3), 140.5(10)	3.34 (m, 1H)	33.6	80.2(3),140.5(10)
4b	3.13 (dd, 16.6,3.1 Hz, 1H)	33.4	108.4(9), 118.3(5), 140.5(10)	3.15 (dd, 16.6,3.2 Hz, 1H)	33.6	108.5(9), 118.4(5), 140.5(10)
5	6.87 (d, 7.4 Hz, 1H)	118.3	33.4(4), 108.4(9)	6.87 (m, 1H)	118.4	33.6(4),108.5(9), 115.5(7)
6	7.52 (dd, 8.4,7.4 Hz, 1H)	136.2	140.5(10),160.9(8)	7.52 (dd, 8.4,7.4 Hz, 1H)	136.3	140.5(10), 160.9(8)
7	6.90 (d, 8.4 Hz, 1H)	115.3	108.4(9), 118.3(5), 160.9(8)	6.90 (m, 1H)	115.5	108.5(9), 118.4(5)
8	-	160.9	-	-	160.9	-
9	-	108.4	-	-	108.5	-
10	-	140.5	-	-	140.5	-
11	-	-	-	3.78 (s, 3H)	55.6	148.0(4')
1'	-	128.4	-	-	130.8	-
2'	7.34 (m, 1H)	128.2	80.4(3), 157.7(4')	6.94 (m, 1H)	114.0	80.2(3),117.6(6')
3'	6.81 (m, 1H)	115.1	128.4(1'), 157.7(4')	-	146.5	-
4'	-	157.7	-	-	148.0	-
5'	6.81 (m, 1H)	115.1	128.4(1'), 157.7(4')	6.95 (m, 1H)	112.0	130.8(1'), 146.5(3')
6'	7.34 (m, 1H)	128.2	80.4(3), 157.7(4')	6.89 (m, 1H)	117.6	80.2(3), 114.0(2'), 148.0(4')

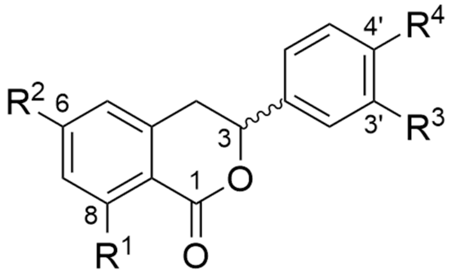

Hydrangenol-8-O-Glc (**1**) R1: O-β-Glc, R2: H, R3: H, R4: OH
Thunberginol G-3‘-O-Glc (**2**) R1: OH, R2: H, R3: O-β-Glc, R4: OH
Phyllodulcin-8-O-Glc (**3**) R1: O-β-Glc, R2: H, R3: OH, R4: OMe
Hydrangenol-4‘-O-Glc (**4**) R1: OH, R2: H, R3: H, R4: O-β-Glc
Phyllodulcin-3’-O-Glc (**5**) R1: OH, R2: H, R3: O-β-Glc, R4: OMe
Thunberginol C-8-O-Glc (**6**) R1: O-β-Glc, R2: OH, R3: H, R4: OH
Thunberginol E-6-O-Glc (**7**) R1: OH, R2: O-β-Glc, R3: OH, R4: OMe
Thunberginol D-6-O-Glc (**8**) R1: OH, R2: O-β-Glc, R3: OH, R4: OH
Thunberginol C (**9**) R1: OH, R2: OH, R3: H, R4: OH
Thunberginol E (**10**) R1: OH, R2: OH, R3: OH, R4: OMe
Thunberginol G (**11**) R1: OH, R2: H, R3: OH, R4: OH
Hydrangenol (**12**) R1: OH, R2: H, R3: H, R4: OH
Phyllodulcin (**13**) R1: OH, R2: H, R3: OH, R4: OMe

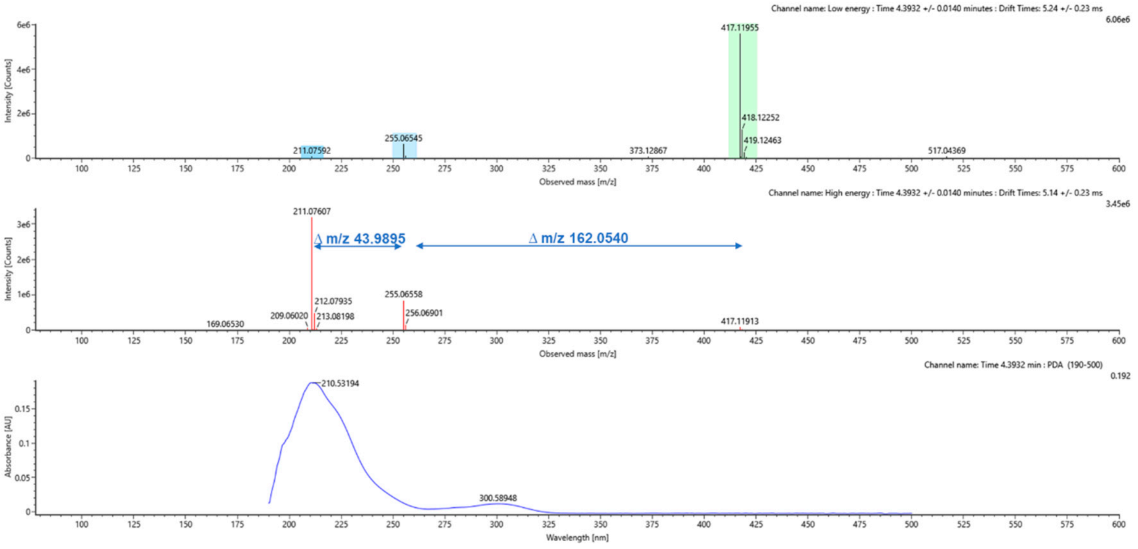

Figure A1: mass and UV-spectra of hydrangenol 8-O-glc (1)

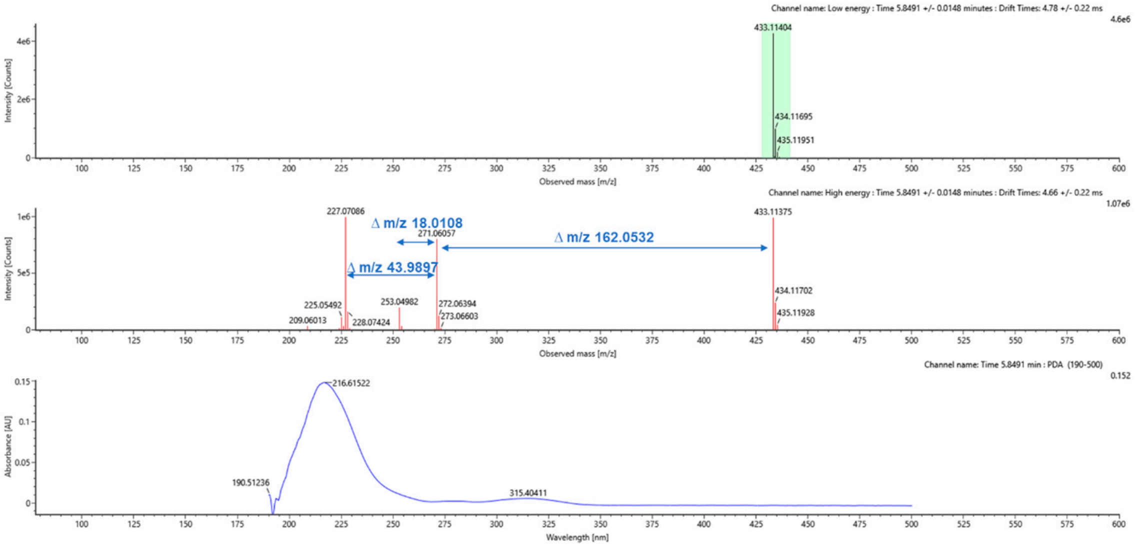

Figure A2: mass and UV-spectra of thunberginol G-3'-O-glc (2)

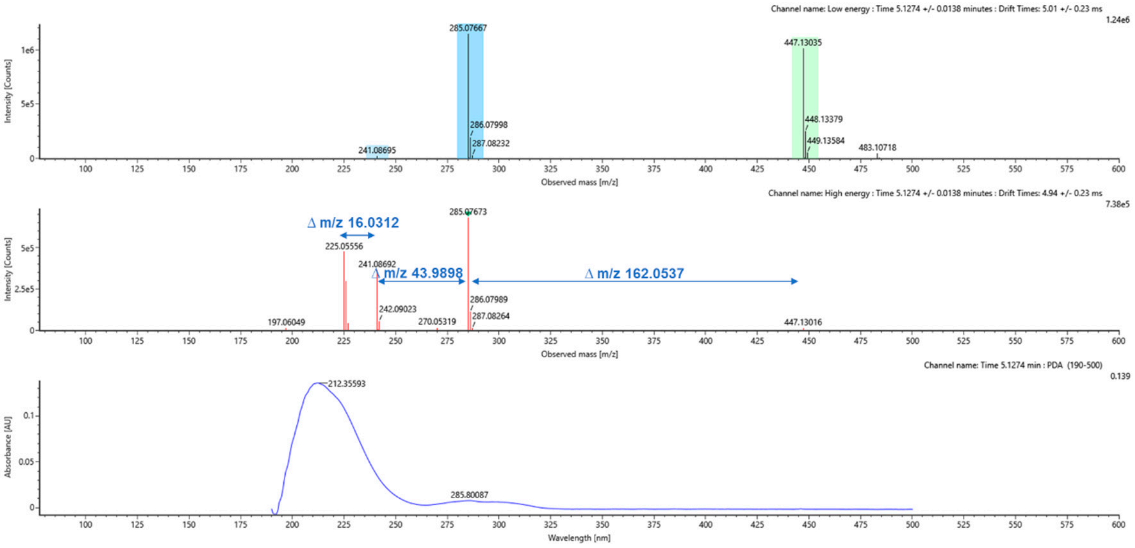

Figure A3: mass and UV-spectra of phyllodulcin 8-O-glc (3)

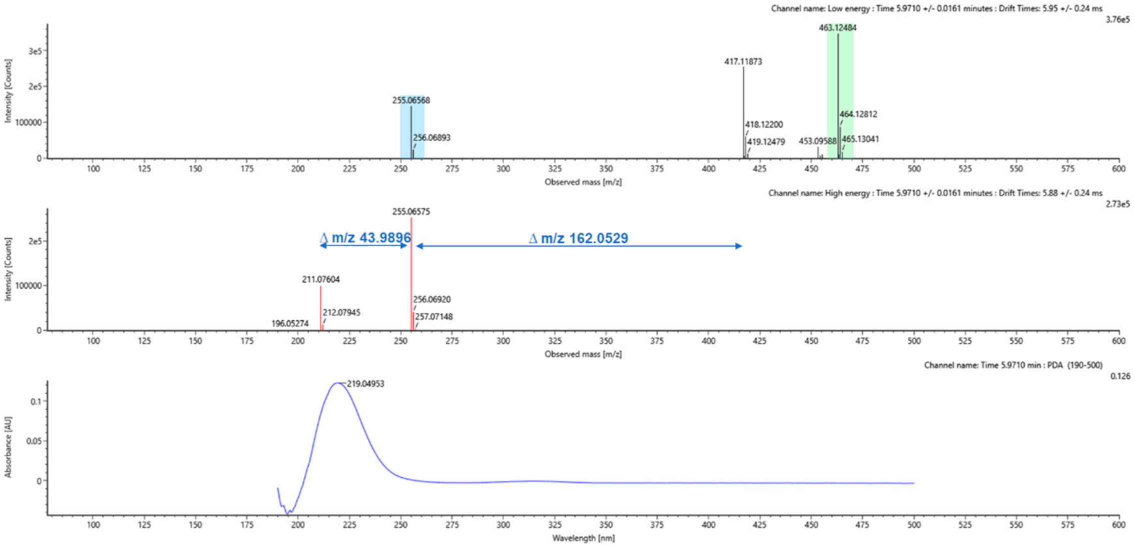

Figure A4: mass and UV-spectra of hydrangenol 4'-O-glc (4)

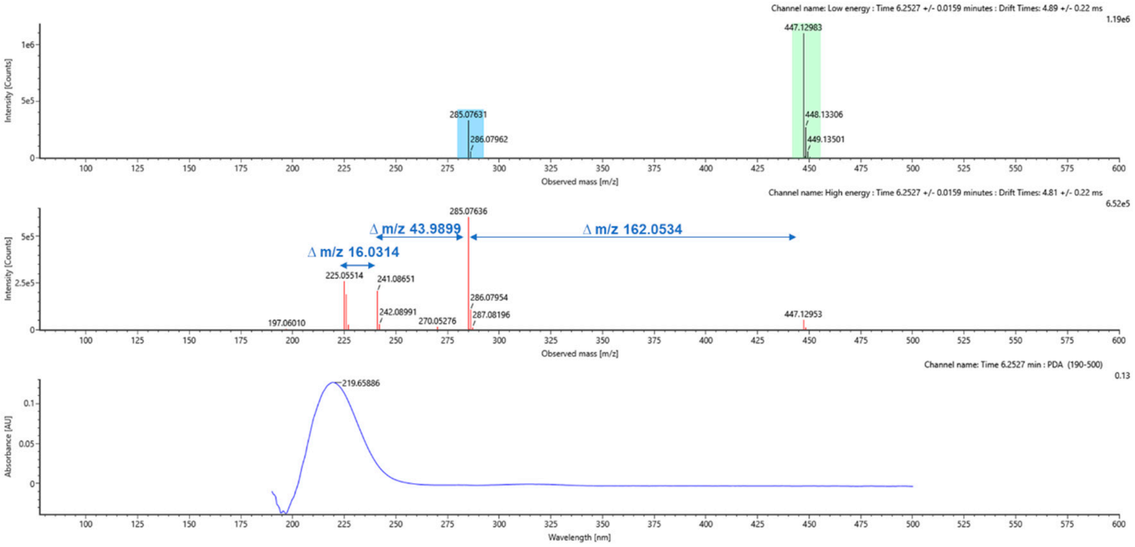

Figure A5: mass and UV-spectra of phyllodulcin 3'-O-glc (5)

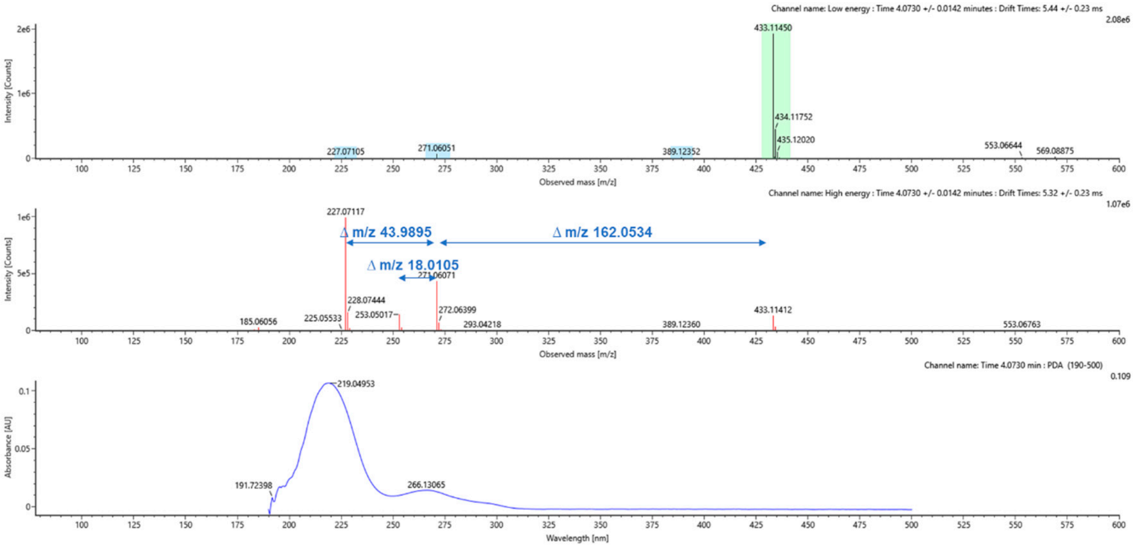

Figure A6: mass and UV-spectra of thunberginol C-8-O-glc (6)

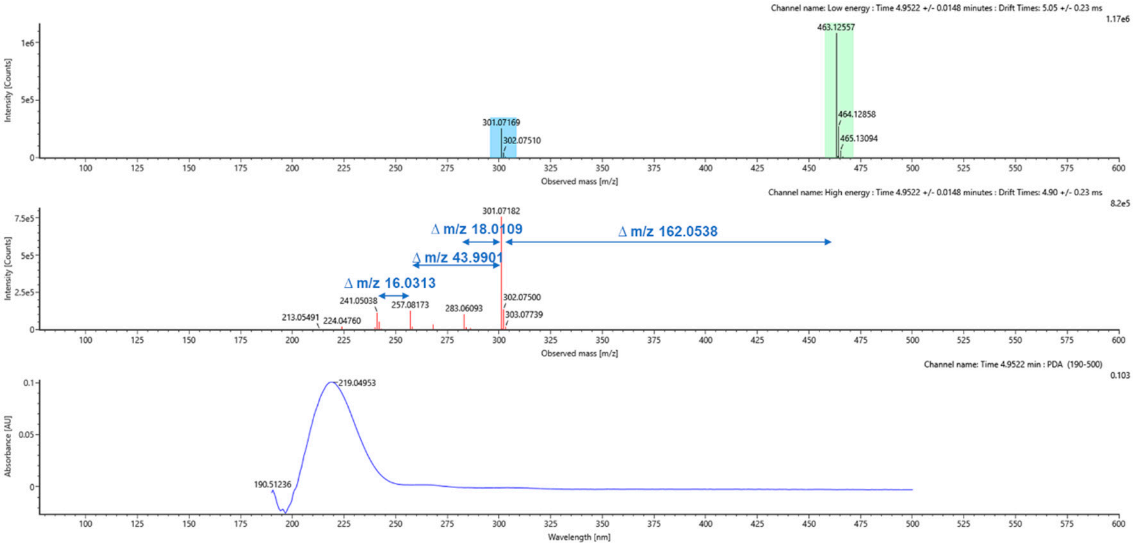

Figure A7: mass and UV-spectra of thunberginol E-6-O-glc (7)

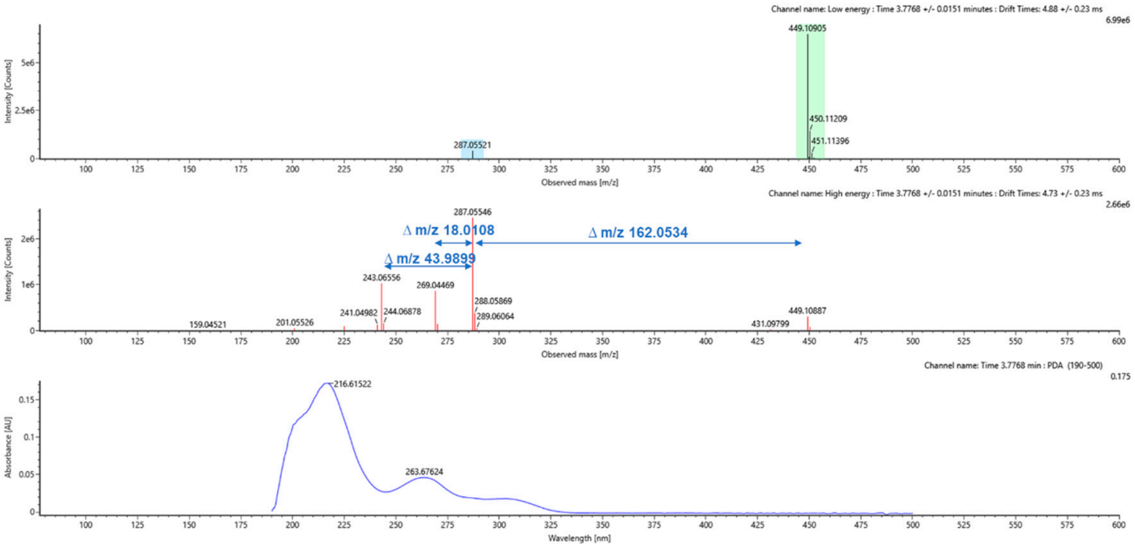

Figure A8: mass and UV-spectra of thunberginol D-6-O-glc (8)

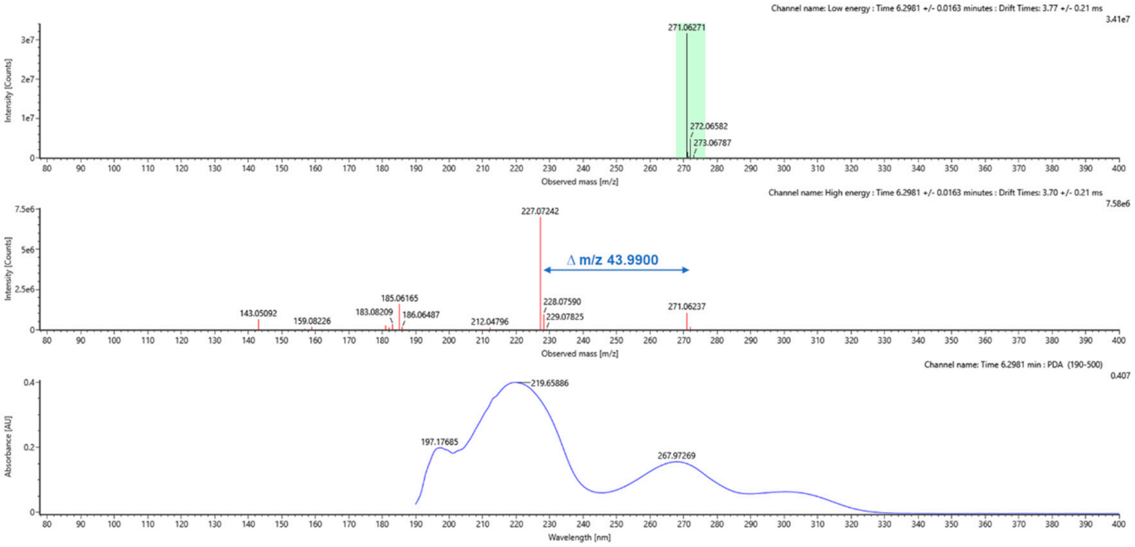

Figure A9: mass and UV spectra of thunberginol C (9)

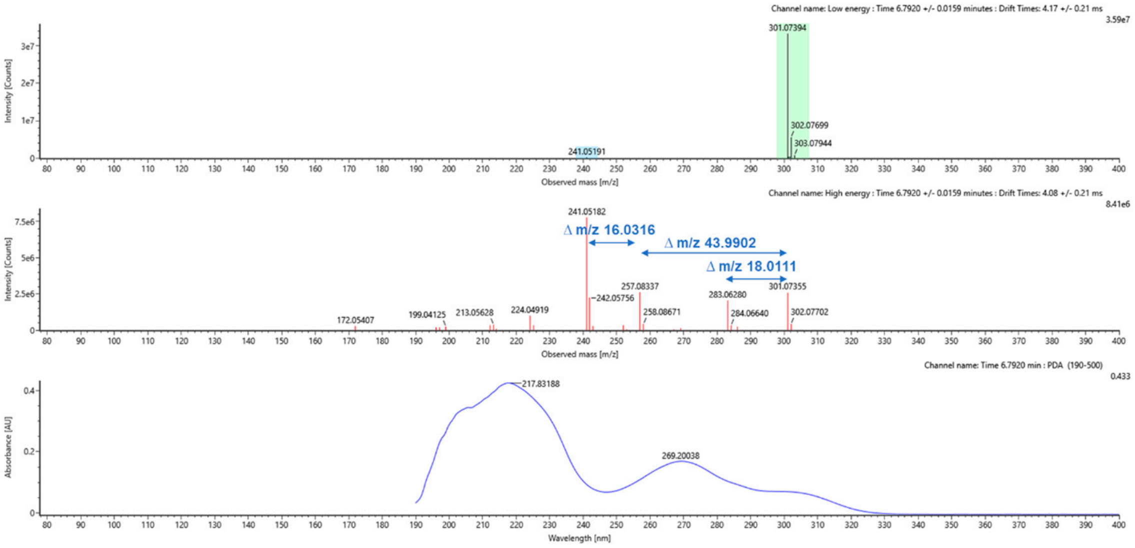

Figure A10: mass and UV spectra of thunberginol E (10)

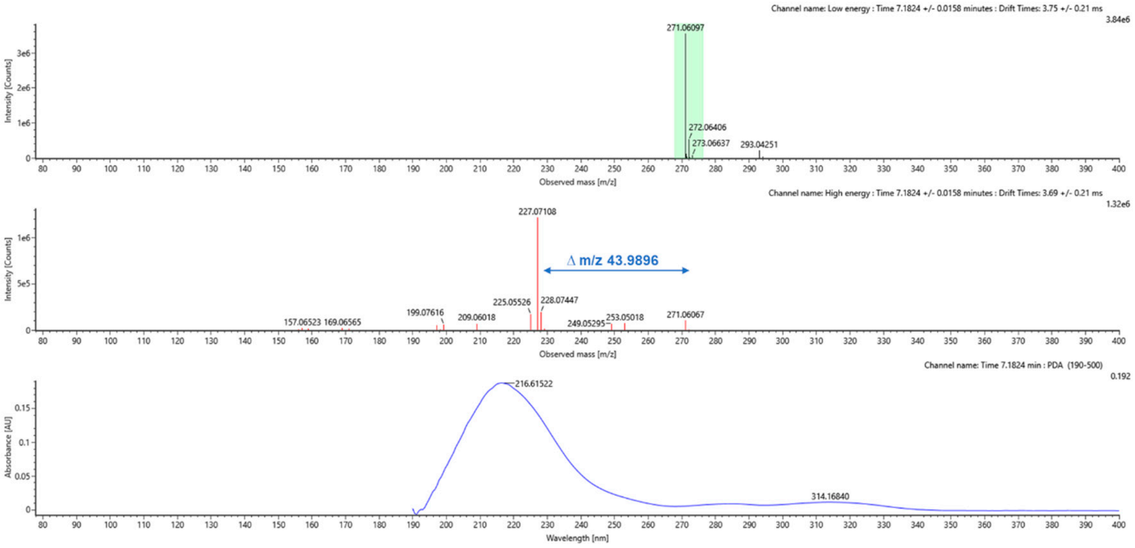

Figure A11: mass and UV-spectra of thunberginol G (11)

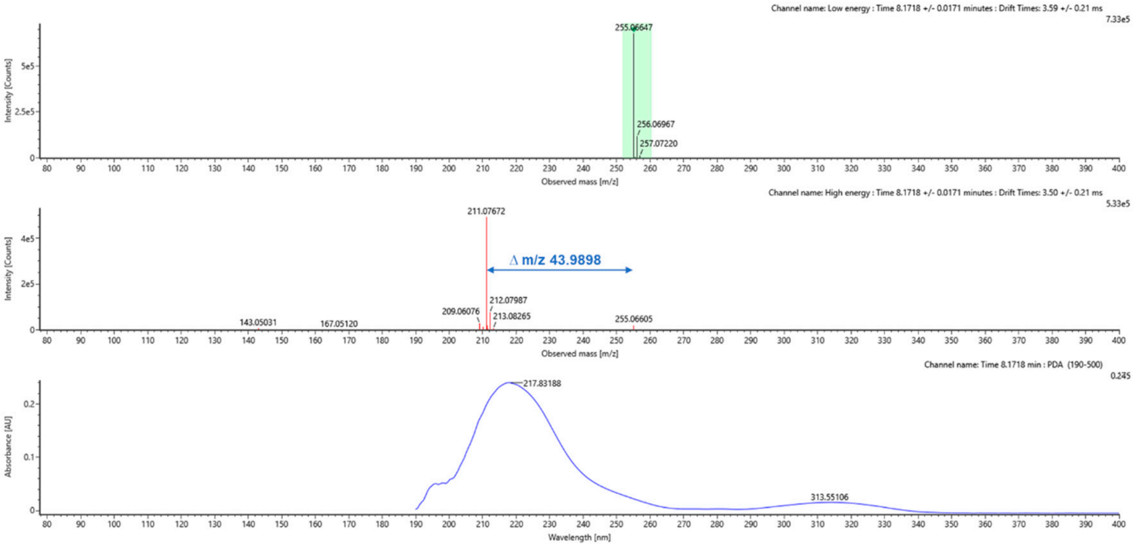

Figure A12: mass and UV-spectra of hydrangenol (12)

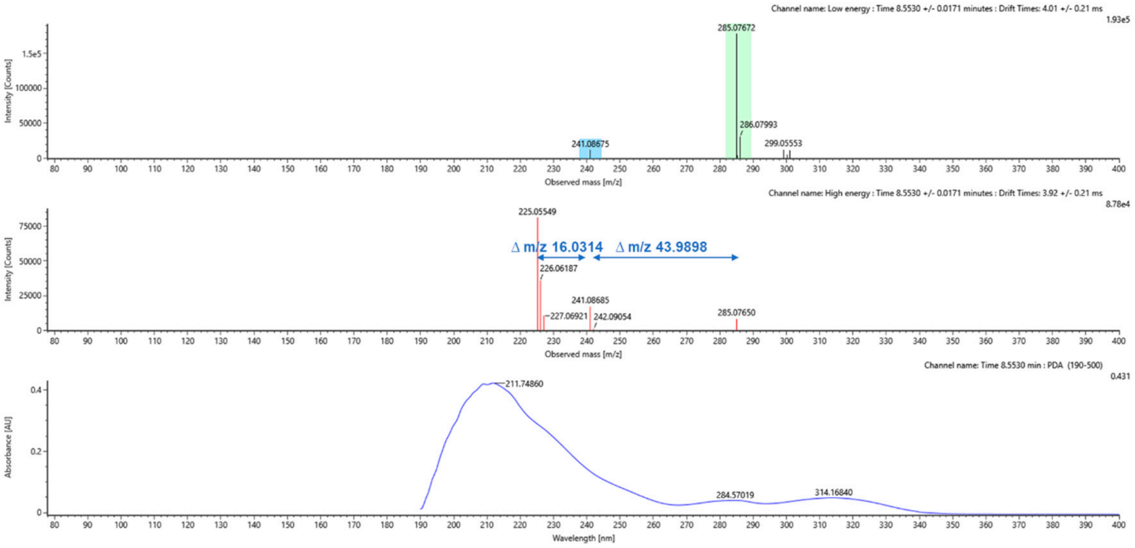

Figure A13: mass and UV-spectra of phyllodulcin (13)
