# Supplementary material for: Separation of Dihydro-Isocoumarins and Dihydro-Stilbenoids from Hydrangea macrophylla ssp. serrata by Use of Counter-Current Chromatography
Source: Molecules. 2022 May 26;27(11):3424. doi: 10.3390/molecules27113424 (PMC9182509; doi:10.3390/molecules27113424)
Supplement: Supplementary file 1 [file molecules-27-03424-s001.zip › supplementalfile_S2_dihydrostilbenic acids.pdf]

Supplemental File S2: NMR and mass spectrometry of dihydrostilbenic acids

Table B1: NMR assignments of cudrabibenzyl A and 2,4-Dihydroxy-6-[2-(4-hydroxyphenyl)ethyl]benzoic acid

	**cudrabibenzyl A**			**2,4-dihydroxy-6-[2-(4-hydroxyphenyl)ethyl]benzoic acid**		
**No**	**δ $_H$ (Multiplicity, *J*, nH)**	**δ $_C$**	**HMBC**	**δ $_H$ (Multiplicity, *J*, nH)**	**δ $_C$**	**HMBC**
1	-	108.8	-	-	109.7	-
2	-	165.8	-	-	166.4	-
3	6.47 (m, 1H)	103	108.8(1), 112.2(5), 162.5(4), 165.8(2)	5.89 (m, 1H)	100.4	107.2(5), 109.7(1), 158.8(4),166.4(2)
4	-	162.5	-	-	158.8	-
5	6.40 (m, 1H)	112.2	40.1(7), 103.0(3), 108.8(1), 162.5(4)	5.88 (m, 1H)	107.2	38.1(7), 100.4(3), 109.7(1)
6	-	148.7	-	-	146.6	-
7a	3.22 (m, 1H)	40.1	38.4(8), 112.2(5), 148.7(6)	3.13 (m, 2H)	38.1	37.1(8), 107.2(5), 109.7(1),133.1(1'), 146.6(6)
7b	3.11 (m, 1H)	40.1	38.4(8), 112.2(5), 148.7(6)	-	-	-
8	2.76 (m, 2H)	38.4	40.1(7), 130.4(2'), 130.4(6'), 134.4(1'),148.7(6)	2.63 (m, 2H)	37.1	38.1(7), 129.1(6'), 129.1(2'),133.1(1'), 146.6(6)
9	-	174.6	-	-	172.4	-
1'	-	134.4	-	-	133.1	-
2'	7.01 (m, 1H)	130.4	38.4(8), 156.3(4')	7.04 (m, 1H)	129.1	37.1(8), 155.0(4’)
3'	6.69 (m, 1H)	116.0	134.4(1'), 156.3(4')	6.65 (m, 1H)	114.8	133.1(1’), 155.0(4’)
4'	-	156.3	-	-	155.0	-
5'	6.69 (m, 1H)	116	134.4(1'), 156.3(4')	6.65 (m, 1H)	114.8	133.1(1’), 155.0(4’)
6'	7.01 (m, 1H)	130.4	38.4(8), 156.3(4')	7.04 (m, 1H)	129.1	37.1(8), 155.0(4’)
A1	4.87 (d, 7.6 Hz, 1H)	101.4	162.5(4)			
A2	3.45 (m, 1H))	74.7	-			
A3	3.49 (m, 1H)	77.8	71.2(A4),74.7(A2)			
A4	3.40 (dd, 9.7,8.5 Hz, 1H)	71.2	62.3(A6)			
A5	3.45 (m, 1H)	78.1	-			
A6a	3.89 (dd, 12.2,2.3 Hz, 1H)	62.3	71.2(A4)			
A6b	3.71 (dd, 12.2,5.4 Hz, 1H)	62.3	78.1(A5)			

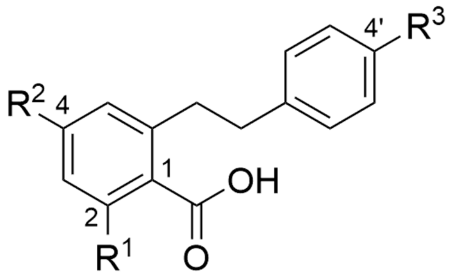

Cubrabibenzyl A (**14**) R1: OH, R2: O-β-Glc, R3: OH
2,4-dihydroxy-6-[2-(4-hydroxyphenyl)ethyl]benzoic acid (**15**) R1: OH, R2: OH, R3: OH

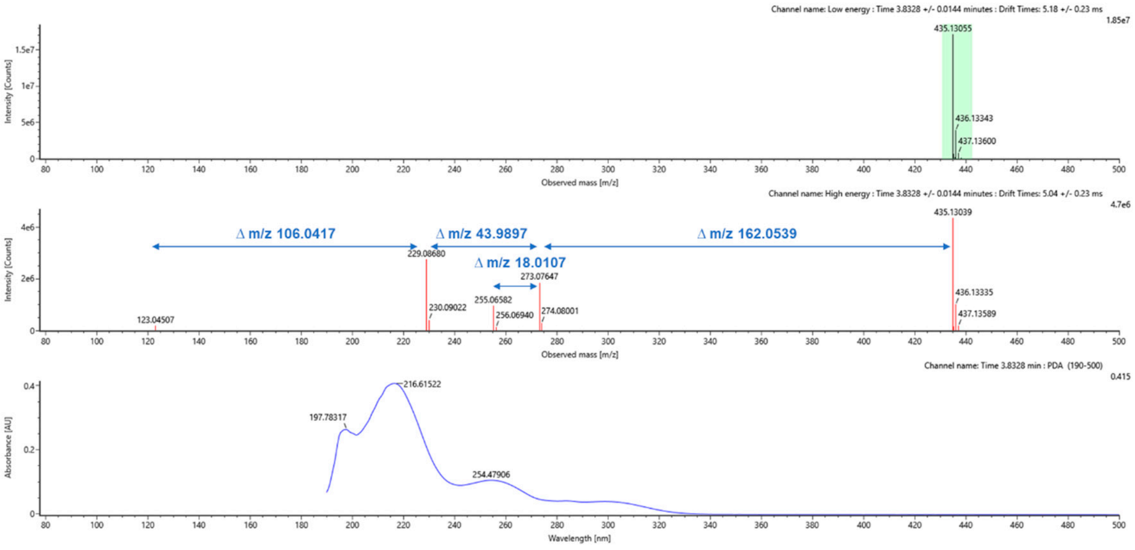

Figure B1: mass and UV-spectra of cudrabibenzyl A (14)

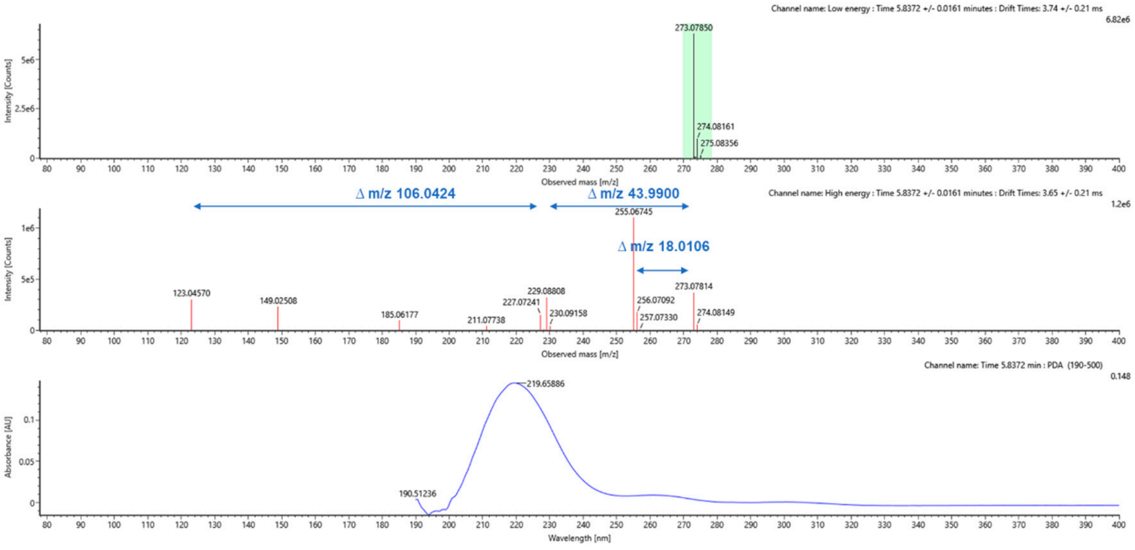

Figure B2: mass and UV spectra of 2,4-dihydroxy-6-[2-(4-hydroxyphenyl)ethyl]benzoic acid (15)
