# Supplementary material for: Separation of Dihydro-Isocoumarins and Dihydro-Stilbenoids from Hydrangea macrophylla ssp. serrata by Use of Counter-Current Chromatography
Source: Molecules. 2022 May 26;27(11):3424. doi: 10.3390/molecules27113424 (PMC9182509; doi:10.3390/molecules27113424)
Supplement: Supplementary file 1 [file molecules-27-03424-s001.zip › supplementalfile_S3_dihydrostilbene and related compound.pdf]

Supplemental File S3: NMR and mass spectrometry of dihydrostilbenes and a related substance

Table C1: NMR assignments of lunularin-4'-O-glucoside (16) and dihydroresveratrol-3-O-glucoside (17)

	**lunularin-4'-O-glucoside (16)**			**dihydroresveratrol-3-O-glucoside (17)**		
**No**	**δ H (Multiplicity, *J*, nH)**	**δ C**	**HMBC**	**δ H (Multiplicity, *J*, nH)**	**δ C**	**HMBC**
1	-	142.9	-	-	143.7	-
2	6.60 (s, 1H)	115.3	37.3(7)	6.34 (s, 1H)	107.1	37.6(7), 101.1(4),109.1(6)
3	-	157.4	-	-	158.5	-
4	6.55 (d, 8.0 Hz1H)	112.8	118.9(6)	6.26 (s, 1H)	101.1	-
5	7.04 (t, 8.0,7.4 Hz, 1H)	129.1	142.9(1), 157.4(3)	-	158.1	-
6	6.61 (d, 7,6 Hz, 1H)	118.9	37.3(7),115.3(2)	6.26 (s, 1H)	109.1	-
7	2.74 (m, 2H)	37.3	36.1(8)	2.7 (m, 2H)	37.6	131.6(1'),143.7(1)
8	2.79 (m, 2H)	36.1	37.3(7)	2.7 (m, 2H)	35.9	129.1(6'), 129.1(2'), 131.6(1')
1'	-	134.8	-	-	131.6	-
2'	7.11 (m, 1H)	129.1	36.1(8), 155.7(4')	7.00 (m, 1H)	129.1	129.1(6'), 155.4(4')
3'	6.92 (m, 1H)	116.0	116.0(5'),134.8(1'), 155.7(4')	6.64 (m, 1H)	115.0	115.0(5'),131.6(1'), 155.4(4')
4'	-	155.7	-	-	155.4	-
5'	6.92 (m, 1H)	116	116.0(3'),134.8(1'), 155.7(4')	6.64 (m, 1H)	115	115.0(3'),131.6(1'), 155.4(4')
6'	7.11 (m, 1H)	129.1	36.1(8), 155.7(4')	7.00 (m, 1H)	129.1	35.9(8), 129.1(2'),155.4(4')
A1	4.81 (d, 7.7 Hz, 1H)	100.6	155.7(4')	4.73 (d, 7.7 Hz, 1H)	100.5	158.5(3)
A2	3.21 (m, 1H)	73.3	100.6(A1)	3.18 (m, 1H)	73.2	100.5(A1)
A3	3.26 (m 1H)	76.7	69.7(A4),73.3(A2)	3.24 (m, 1H)	76.7	69.7(A4), 73.2(A2)
A4	3.15 (m, 1H)	69.7	-	3.16 (m, 1H)	69.7	-
A5	3.3 (m, 1H)	77.0	-	3.26 (m, 1H)	77	-
A6a	3.68 (m, 1H)	60.7	-	3.68 (m, 1H)	60.6	-
A6b	3.46 (m, 1H)	60.7	-	3.48 (m, 1H)	60.6	-

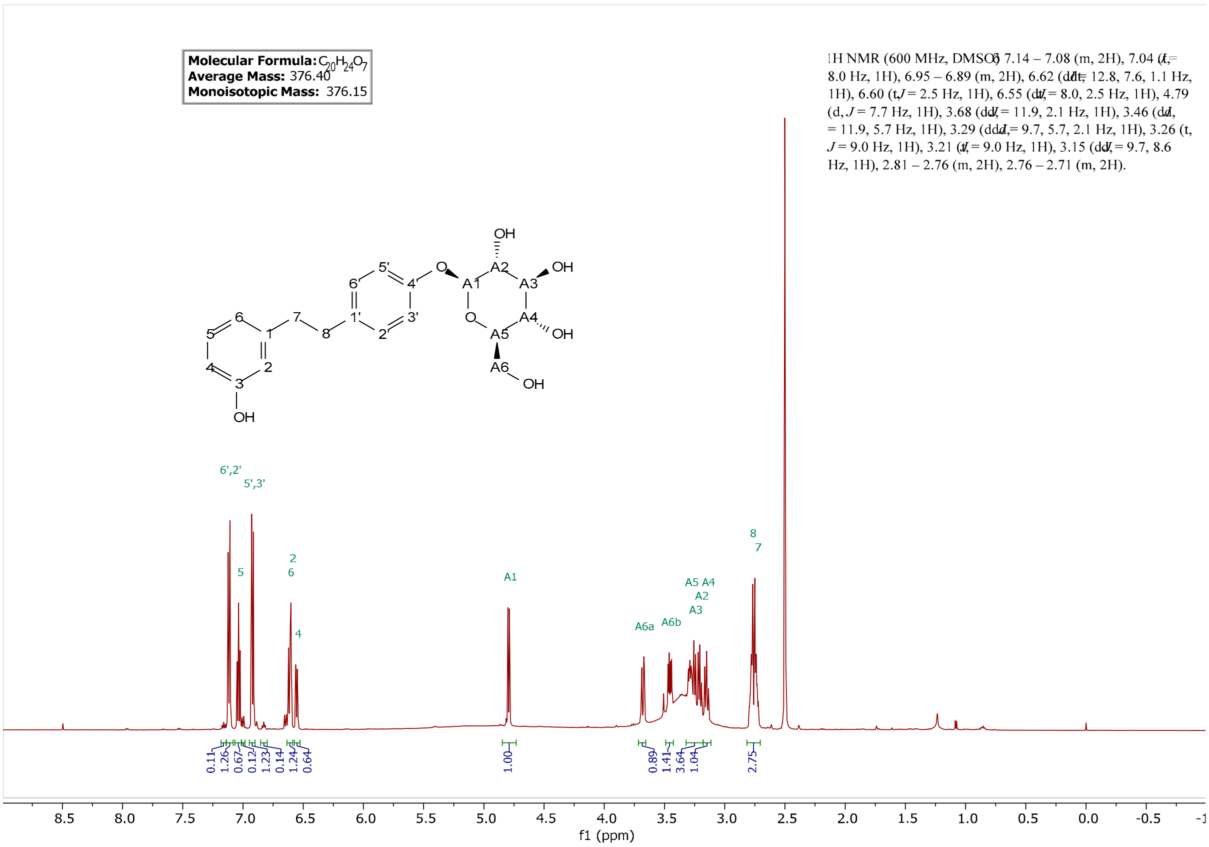

Figure C1: ^{1}H NMR spectrum (600 MHz, DMSO) of lunularin-4'-O-glucoside (16)

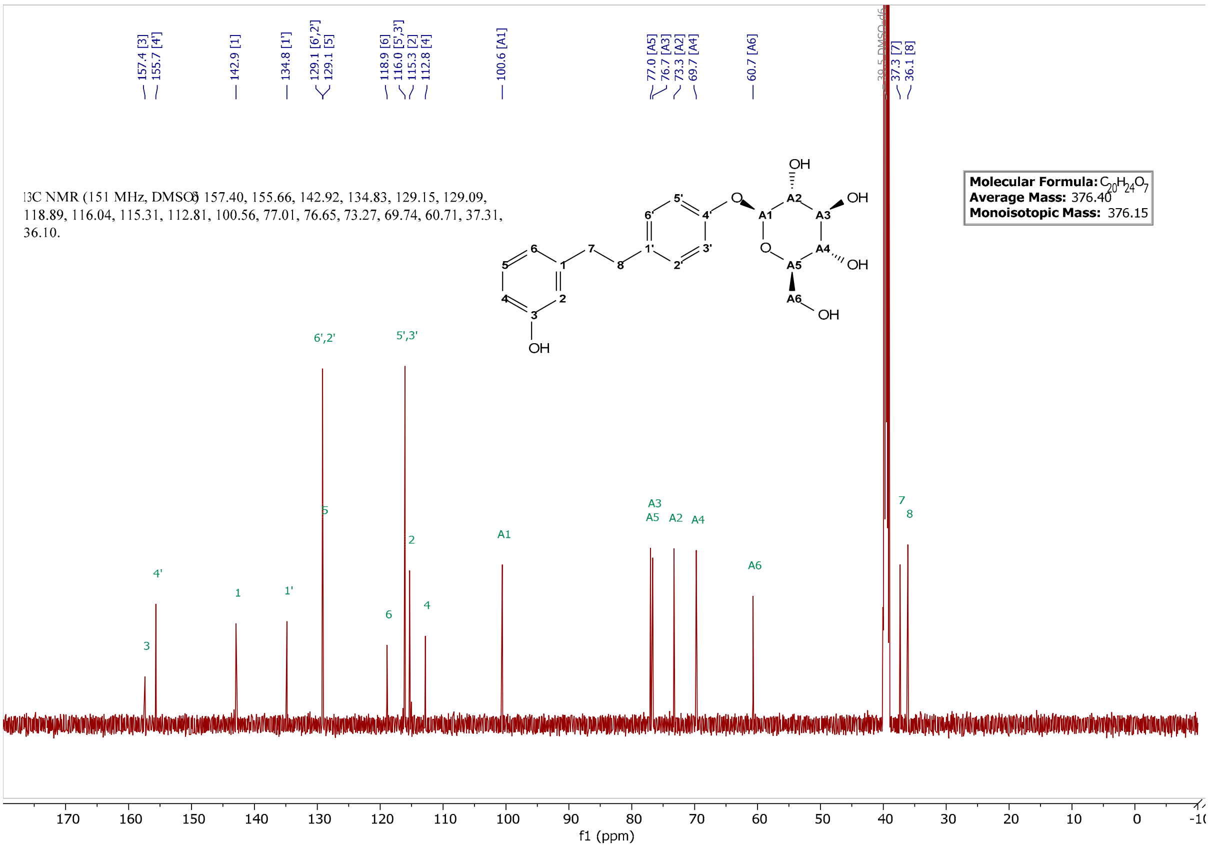

Figure C2: ^{13}C NMR spectrum (151 MHz, DMSO) of lunularin-4'-O-glucoside (16)

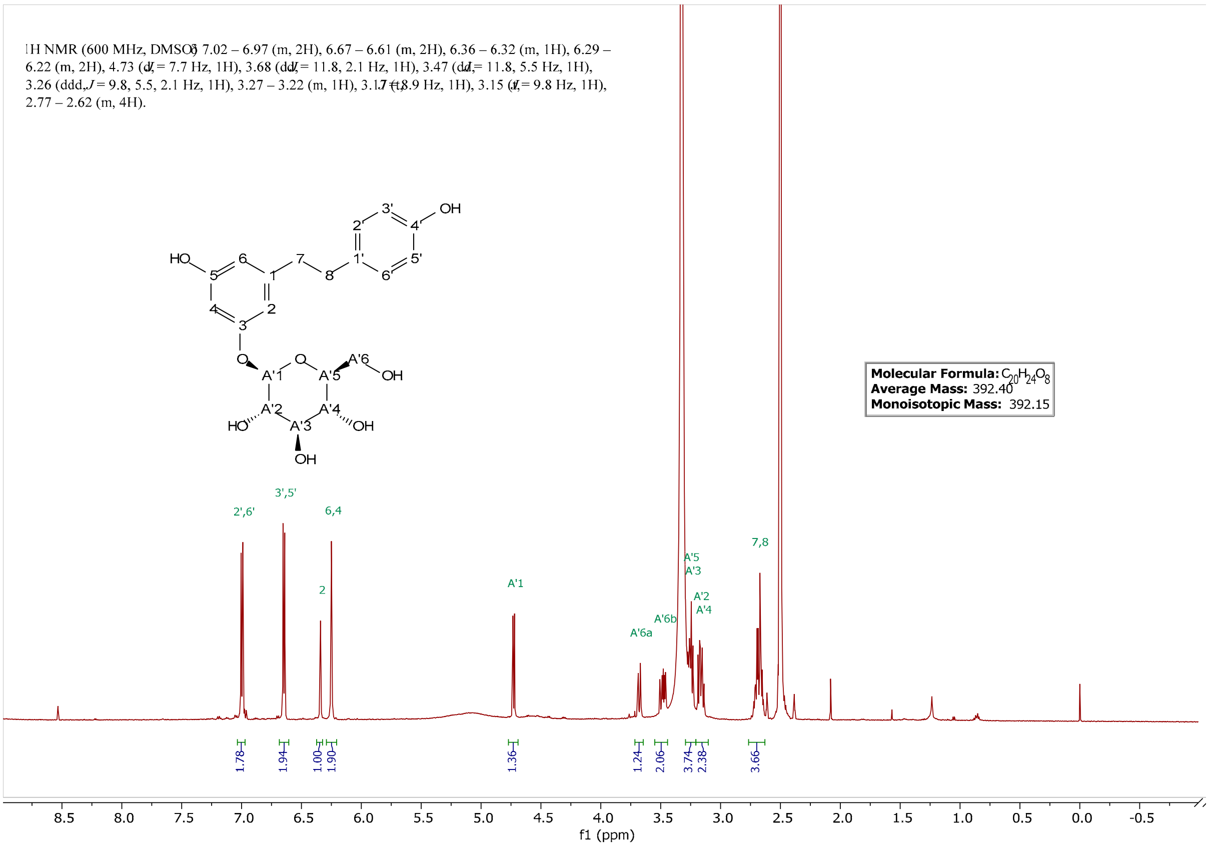

Figure C3: ^{1}H NMR spectrum (600 MHz, DMSO) of dihydroresveratrol-3-O-glucoside (17)

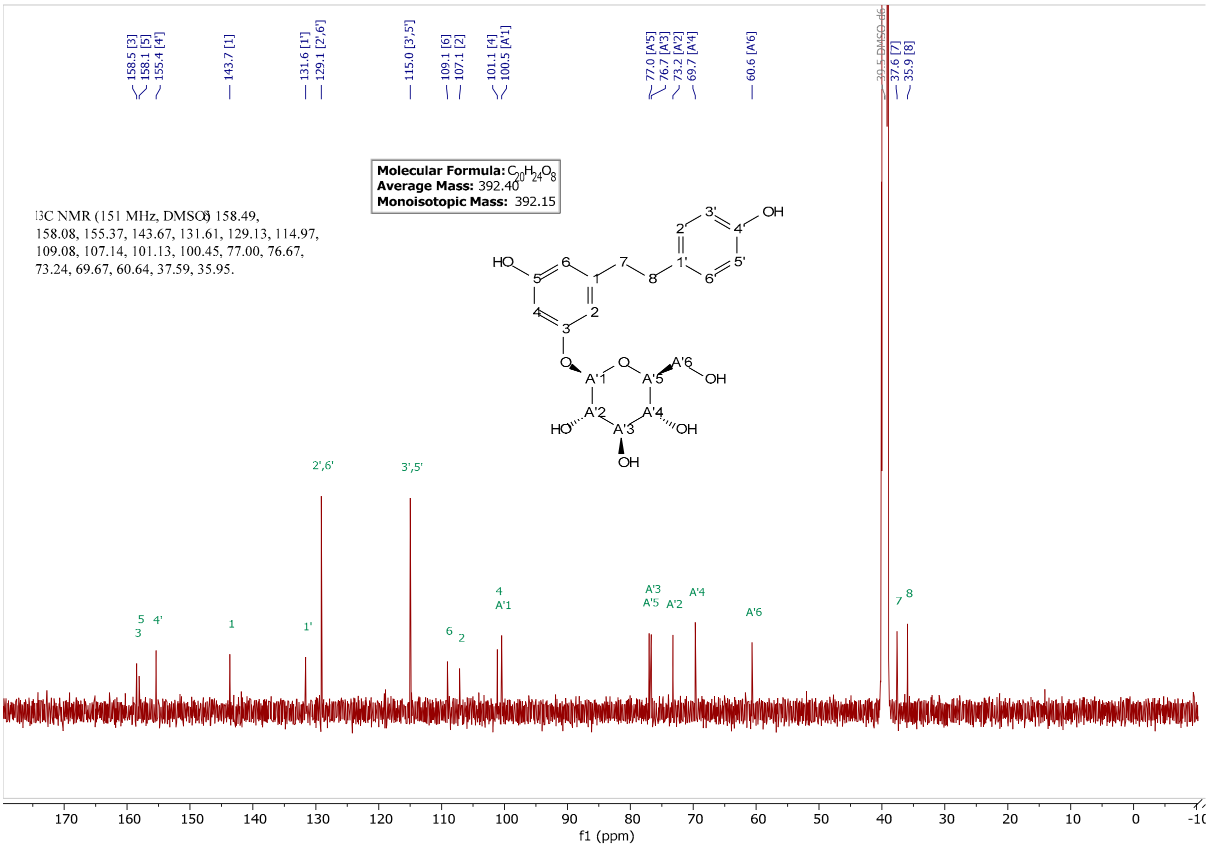

Figure C4: ^{13}C NMR spectrum (151 MHz, DMSO) of dihydroresveratrol-3-O-glucoside (17)

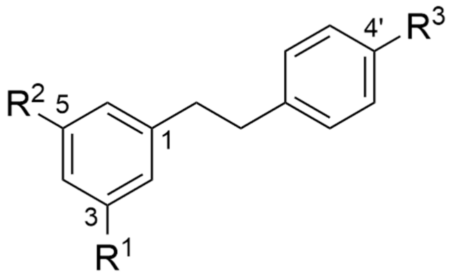

Lunularin 4'-O-glc (**16**) R1: OH, R2: H, R3: O-β-Glc
dihydroresveratrol 3-O-glc (**17**) R1: OH, R2: O-β-Glc, R3: OH

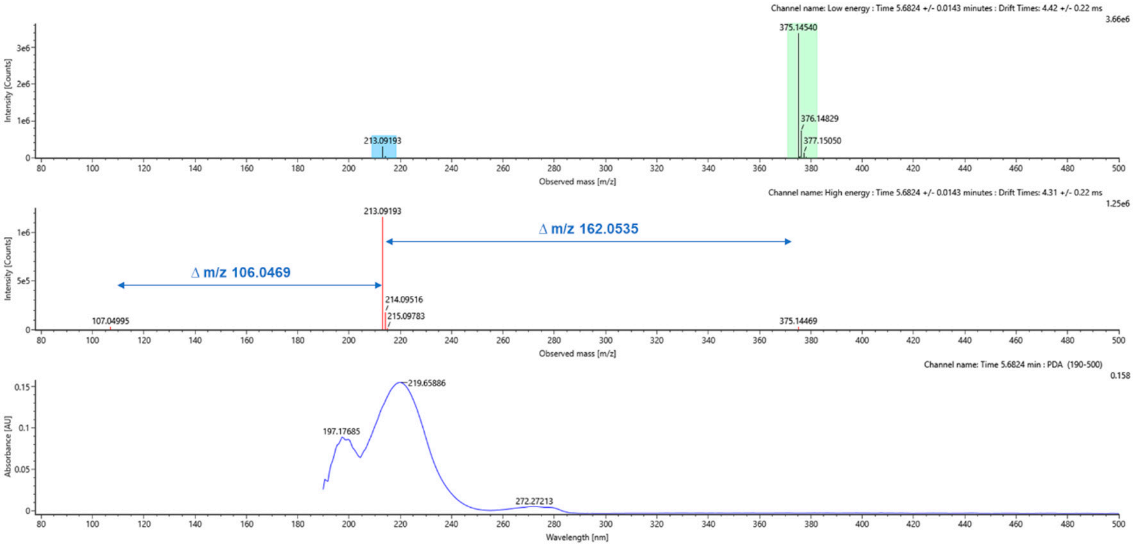

Figure C5: mass and UV-spectra of lunularin-4'-O-glucoside (16)

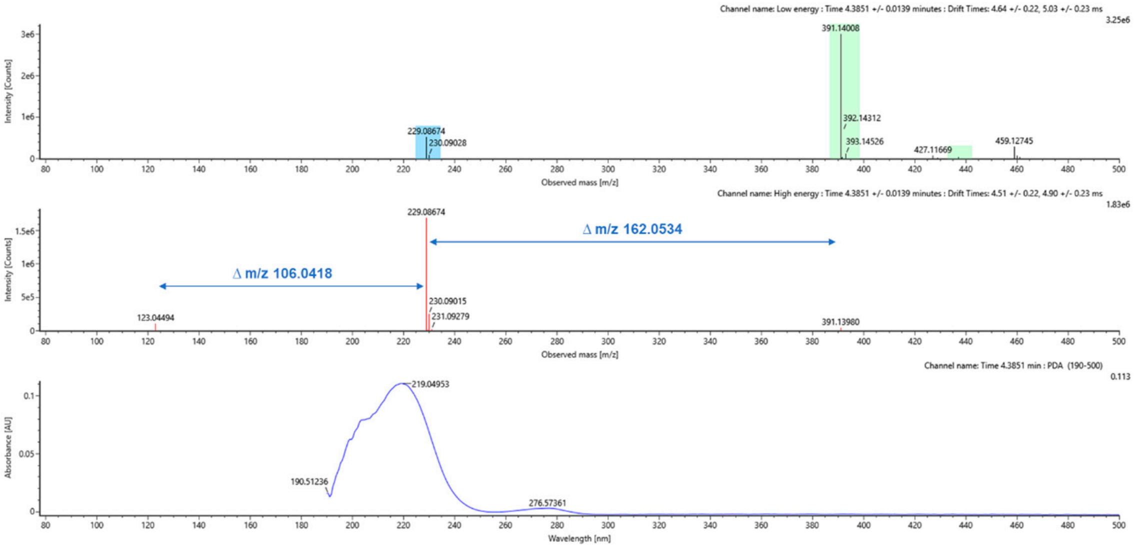

Figure C6: mass and UV spectra of dihydroresveratrol-3-O-glucoside (17)

Table C2: NMR assignments of 4-hydroxy-8-[4-glucopyranosyl]hydroxyphenyl]octane-2,6-dione (18)

No	δ_H (Multiplicity, *J*, nH)	δ_C	HMBC
1	2.08 (s, 3H)	30.5	50.7(3), 207.4(2)
2	-	207.4	-
3	2.47 (m, 2H)	50.7	49.9(5), 63.5(4), 207.4(2)
4	4.29 (tt, 7.7,5.5 Hz, 1H)	63.5	49.9(5), 50.7(3), 207.4(2),208.5(6)
5	2.47 (m, 2H)	49.9	50.7(3), 63.5(4), 208.5(6)
6	-	208.5	-
7	2.72 (m, 2H)	44.4	134.4(1'), 208.5(6)
8	2.72 (m, 2H)	28	44.4(7),129.0(2'), 129.0(6'), 134.4(1'), 208.5(6)
1'	-	134.4	-
2'	7.12 (m, 1H)	129	28.0(8), 155.7(4')
3'	6.94 (m, 1H)	116.2	134.4(1'), 155.7(4')
4'	-	155.7	-
5'	6.94 (m, 1H)	116.2	135.4(1'), zz155.7(4')
6'	7.12 (m, 1H)	129	28.0(8), 155.7(4')
A1	4.77 (d, 7.6 Hz, 1H)	100.6	155.7(4')
A2	3.20 (m, 1H)	73.3	76.6(A3), 100.6(A1)
A3	3.25 (m, 1H)	76.6	69.8(A4), 73.3(A2)
A4	3.14 (m, 1H)	69.8	60.7(A6), 76.6(A3)
A5	3.29 (m, 1H)	77	-
A6a	3.66 (m, 1H)	60.7	69.8(A4)
A6b	3.45 (m, 1H)	60.7	77.0(A5)

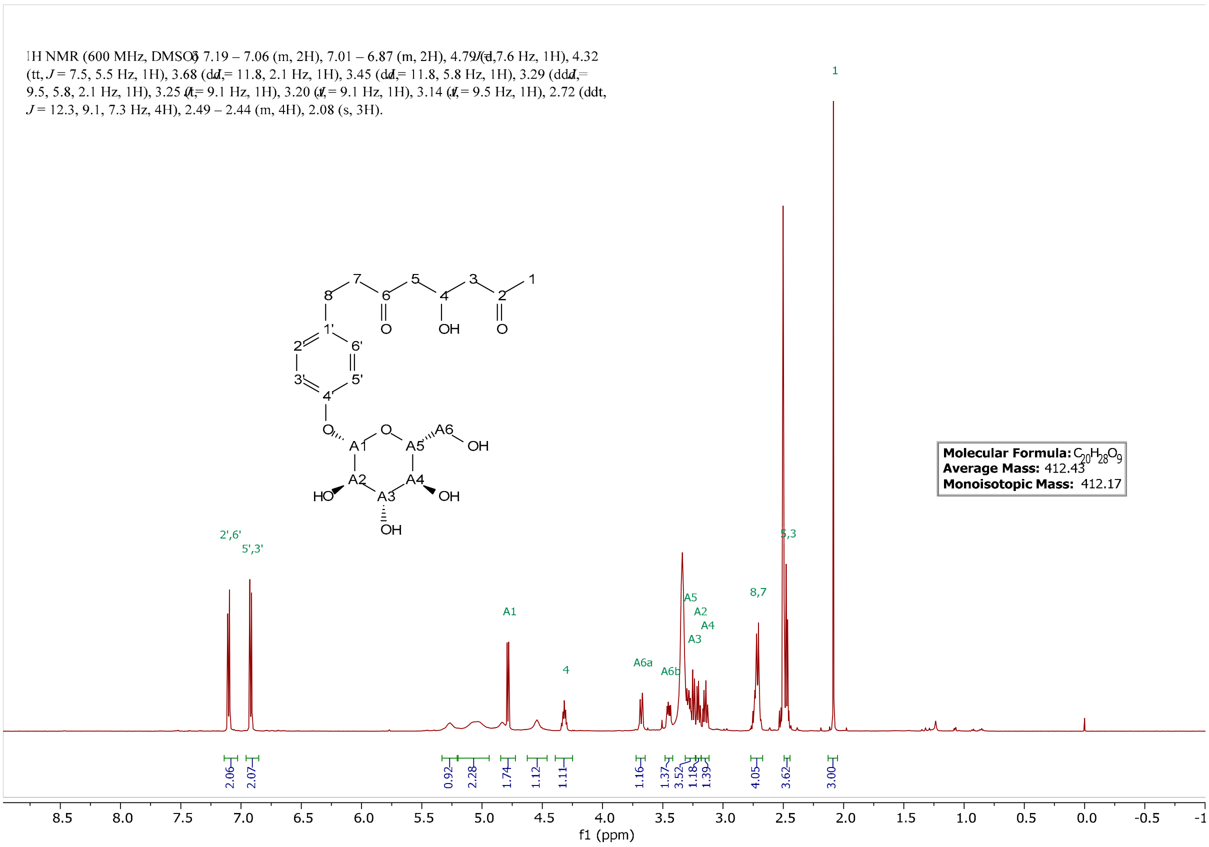

Figure C7: ^{1}H NMR spectrum (600 MHz, DMSO) of 4-hydroxy-8-[4-glucopyranosyl]hydroxyphenyl]octane-2,6-dione (18)

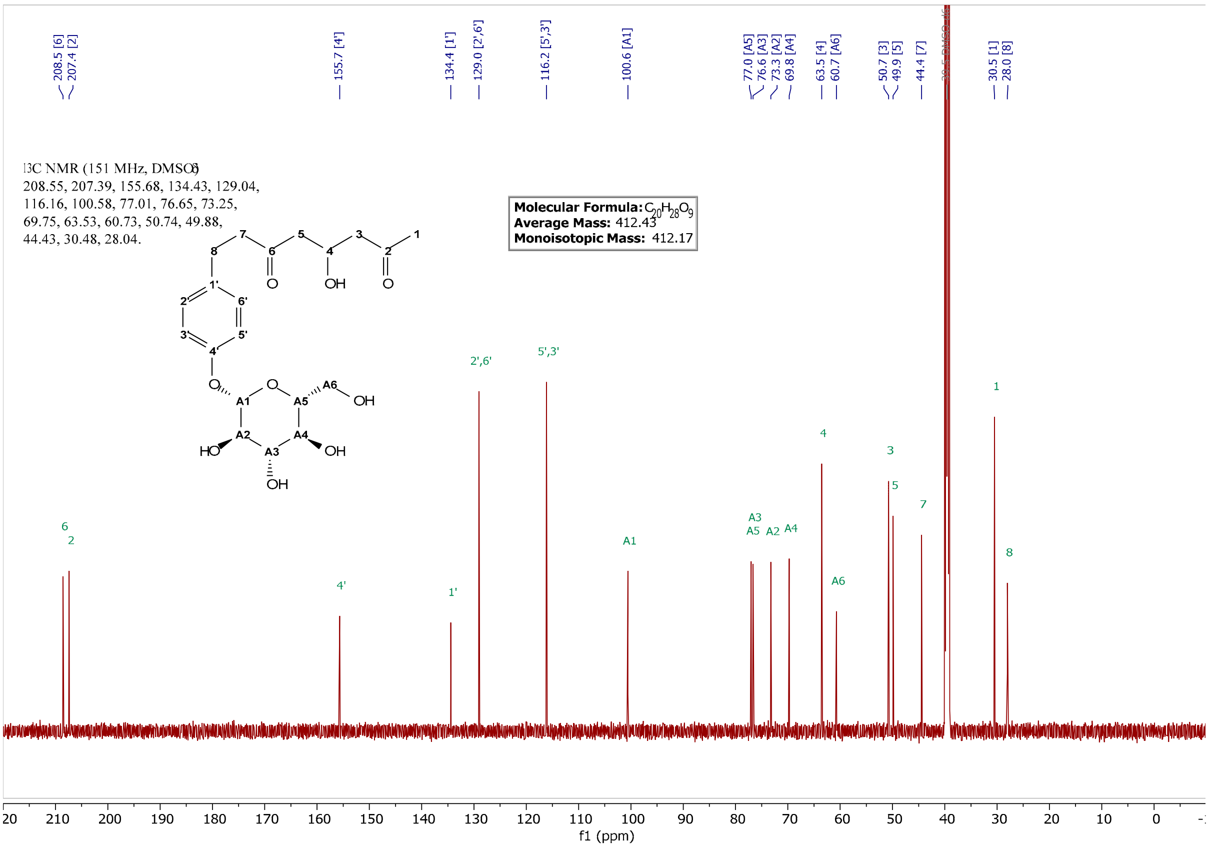

Figure C8: ^{13}C NMR spectrum (151 MHz, DMSO) of 4-hydroxy-8-[4-glucopyranosyl]hydroxyphenyl]octane-2,6-dione (18)

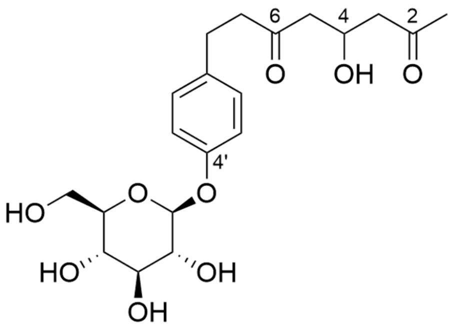

4-hydroxy-8-[4-glucopyranosyl]hydroxyphenyl]octane-2,6-dione (**18**)

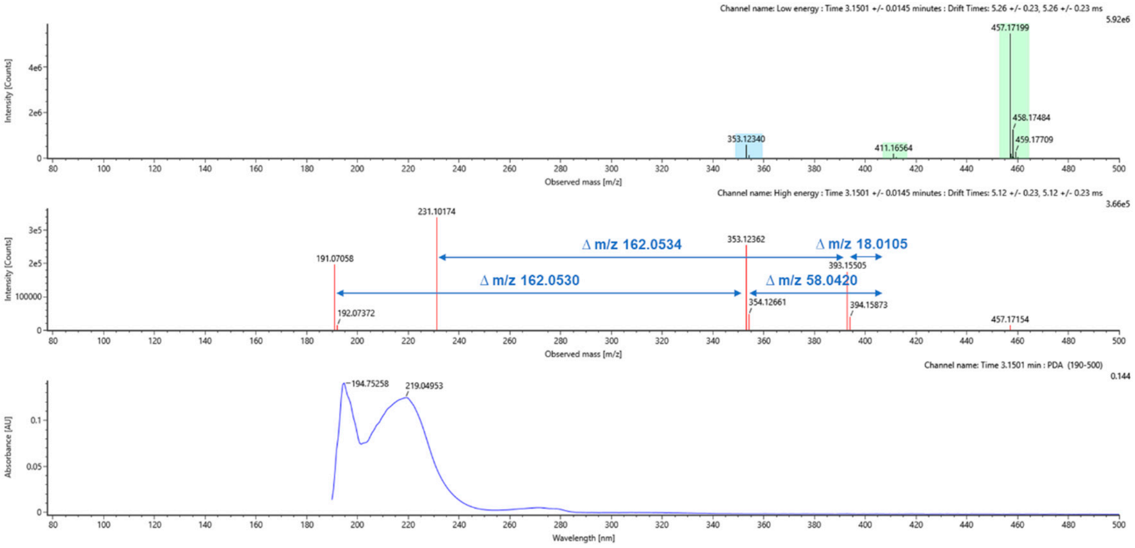

Figure C9: mass and UV-spectra of 4-hydroxy-8-[4-glucopyranosyl]hydroxyphenyl]octane-2,6-dione (18)
