# Supplementary material for: Separation of Dihydro-Isocoumarins and Dihydro-Stilbenoids from Hydrangea macrophylla ssp. serrata by Use of Counter-Current Chromatography
Source: Molecules. 2022 May 26;27(11):3424. doi: 10.3390/molecules27113424 (PMC9182509; doi:10.3390/molecules27113424)
Supplement: Supplementary file 1 [file molecules-27-03424-s001.zip › supplementalfile_S4_taxiphyllin.pdf]

Supplemental File S4: NMR and mass spectrometry of taxiphyllin

Table D1:NMR assignments taxiphyllin (19)

No	δ H (Multiplicity, *J*, nH)	δ C	HMBC
1	-	118.9	-
2	5.89 (s, 1H)	66	100.0(A1), 118.9(1), 123.5(1'),129.3(2'), 129.3(6')
7	9.82 (s, 1H)	-	-
1'	-	123.5	-
2'	7.35 (m, 1H)	129.3	66.0(2), 158.5(4')
3'	6.83 (m, 1H)	115.5	123.5(1'), 158.5(4')
4'	-	158.5	-
5'	6.83 (m, 1H)	115.5	123.5(1'), 158.5(4')
6'	7.35 (m, 1H)	129.3	66.0(2), 158.5(4')
A1	4.11 (d, 7.1, 1H)	100	66.0(2)
A2	3.05 (m, 1H)	73	-
A3	3.05 (m, 1H)	76.5, 77.1	-
A4	3.05 (m, 1H)	69.8	-
A5	3.05 (m, 1H)	76.5, 77.1	-
A6a	3.67 (m, 1H)	61.1	69.8(A4)
A6b	3.52 (m, 1H)	61.1	-

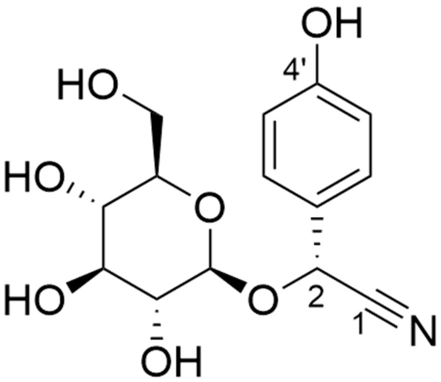

Taxiphyllin (19)

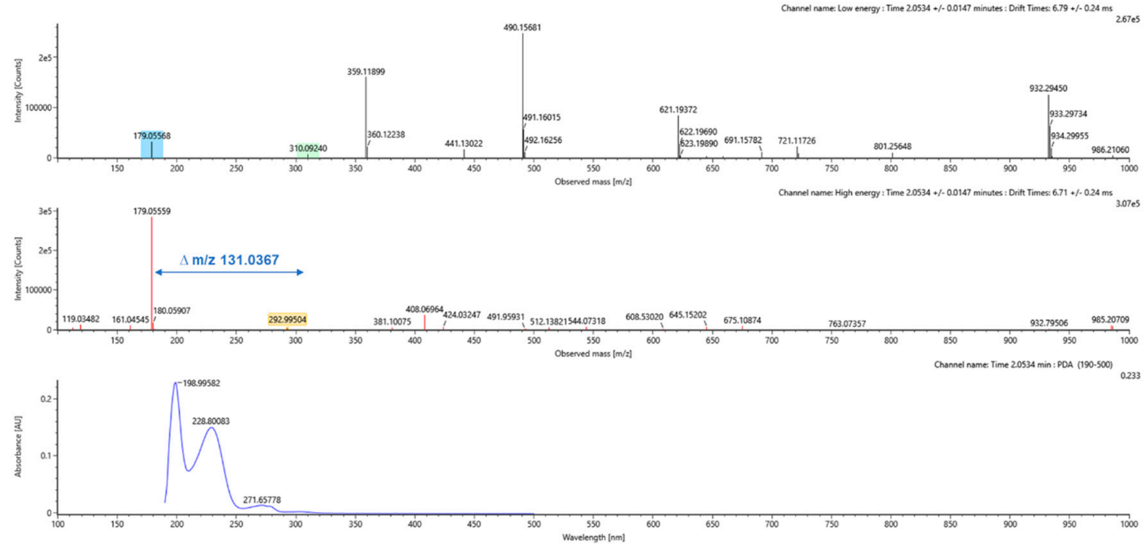

Figure D1: mass and UV-spectra of taxiphyllin (19)
