# Supplementary material for: Separation of Dihydro-Isocoumarins and Dihydro-Stilbenoids from Hydrangea macrophylla ssp. serrata by Use of Counter-Current Chromatography
Source: Molecules. 2022 May 26;27(11):3424. doi: 10.3390/molecules27113424 (PMC9182509; doi:10.3390/molecules27113424)
Supplement: Supplementary file 1 [file molecules-27-03424-s001.zip › supplementalfile_S5_secoiridoid glycosides.pdf]

Supplemental File S5: NMR and mass spectrometry of secoiridoid glycosides

Table E1: NMR assignments of deoxyloganic acid (20)

No	δ $_H$ (Multiplicity, *J*, nH)	δ $_C$	HMBC
1	5.07 (m, 1H)	95.9	33.8(5),98.7(A1), 150.6(3)
3	7.34 (s, 1H)	150.6	33.8(5),95.9(1), 111.4(4), 168.1(11)
4	-	111.4	-
5	2.74 (td, 7.4, 7.2 Hz, 1H)	33.8	31.9(6), 47.2(9),95.9(1), 111.4(4), 150.6(3)
6a	2.09 (m, 1H)	31.9	-
6b	1.31 (m, 1H)	31.9	32.6(7), 33.8(5), 111.4(4)
7a	1.79 (m 1H)	32.6	-
7b	1.11 (m 1H)	32.6	20.5(10)
8	1.88 (m, 1H)	34.8	20.5(10), 32.6(7), 47.2(9), 95.9(1)
9	1.63 (dt, 8.2,6.2 Hz,1H)	47.2	20.5(10), 31.9(6), 33.8(5), 34.8(8), 95.9(1), 111.4(4)
10	1.02 (d, 6.8 Hz, 3H)	20.5	32.6(7), 34.8(8),47.2(9)
11	-	168.1	-
A1	4.49 (d, 7.8 Hz, 1H)	98.7	95.9(1)
A2	2.98 (m, 1H)	73.1	76.8(A3), 95.9(A1)
A3	3.17 (m, 1H)	76.8	-
A4	3.04 (m, 1H)	70.1	-
A5	3.17 (m, 1H)	77.3	-
A6'	3.68 (m, 1H)	61.2	-
A6"	3.44 (m, 1H)	61.2	-

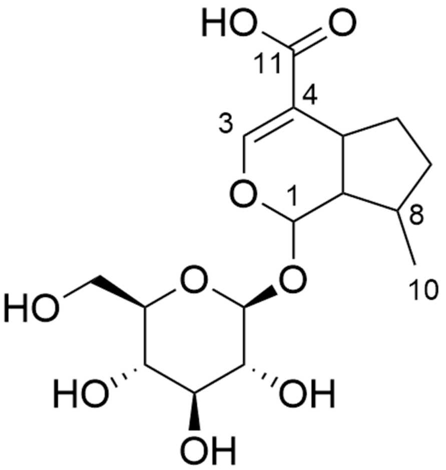

deoxyloganic acid (20)

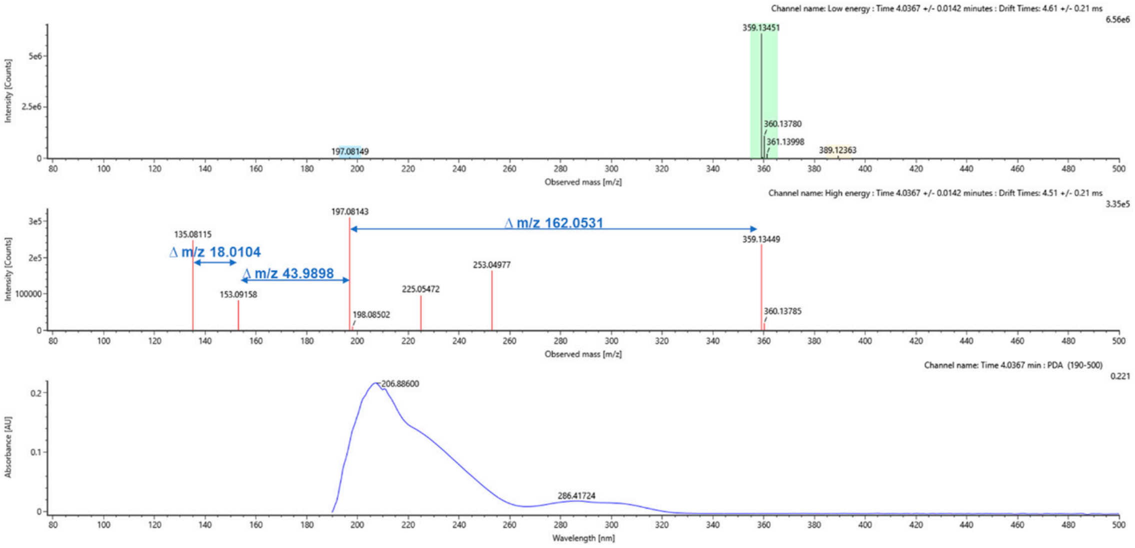

Figure E1: mass and UV-spectra of deoxyloganic acid (20)

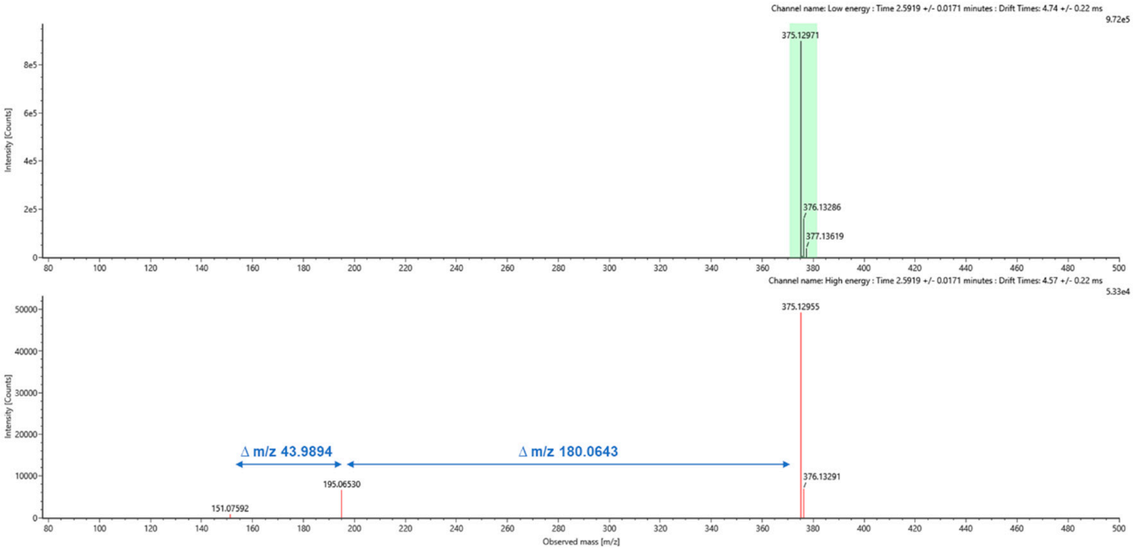

Figure E2: mass spectrum of loganic acid (21)

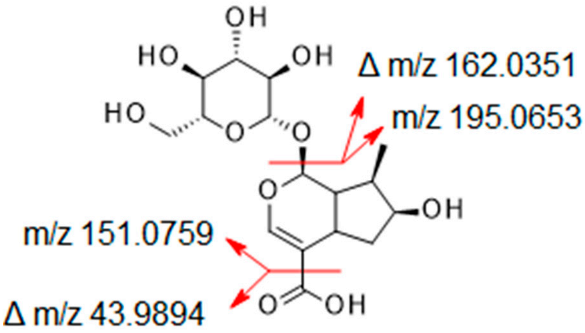

Figure E3: fragmentation loganic acid (21)

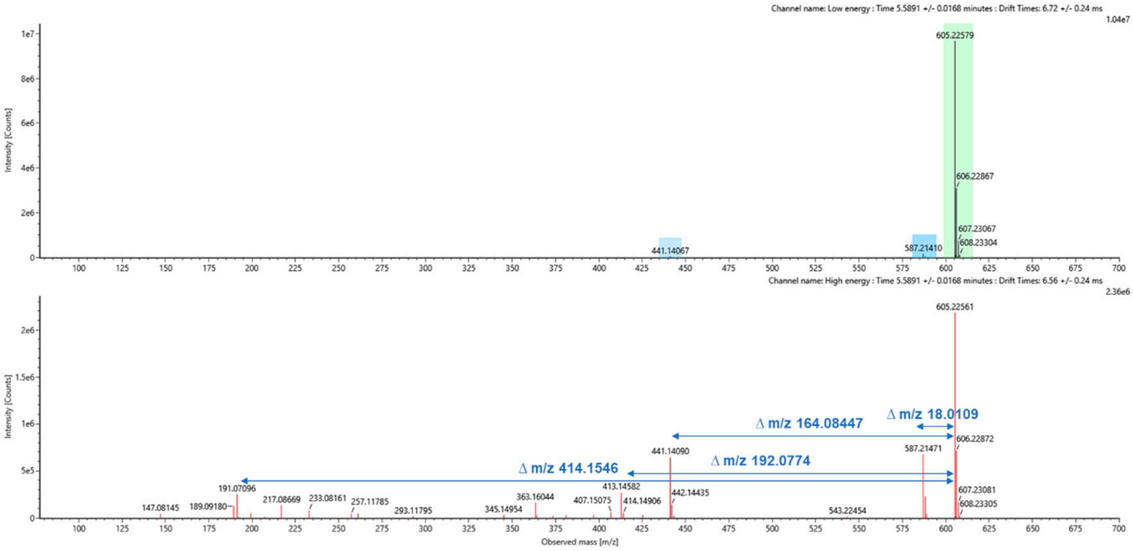

Figure E4: mass spectrum of hydramacroside B (22)

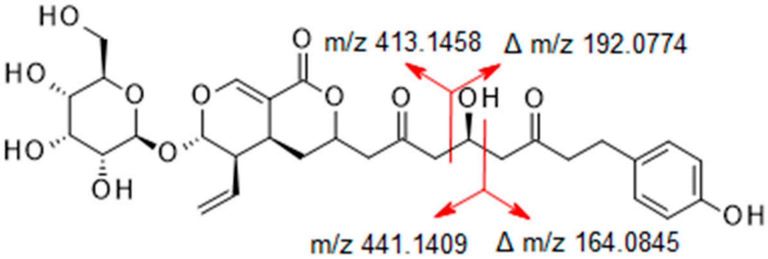

Figure E5: fragmentation hydramacroside B (22)

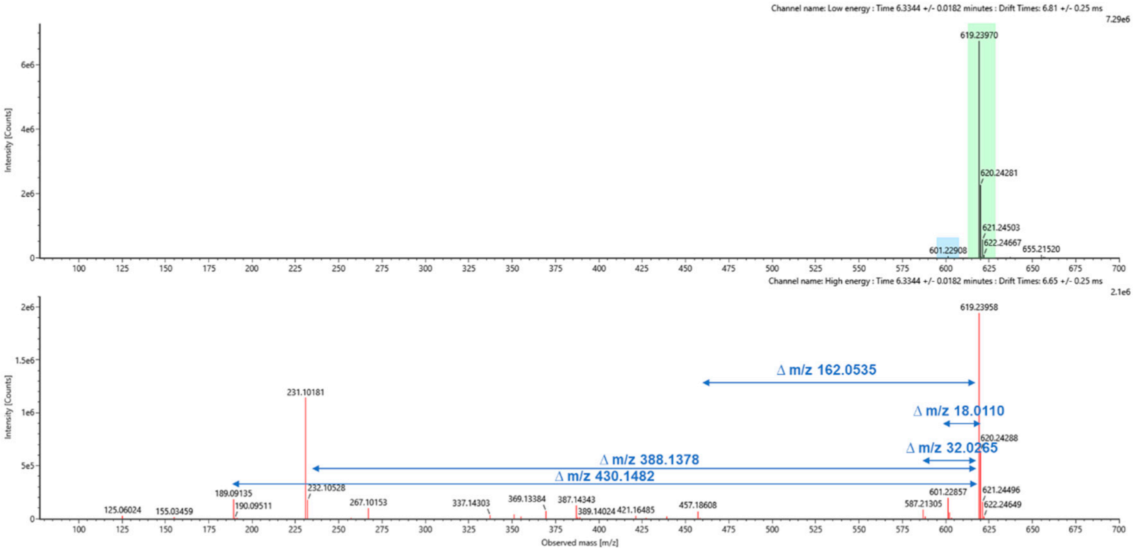

Figure E6: mass spectrum of hydrangenoside A (23)

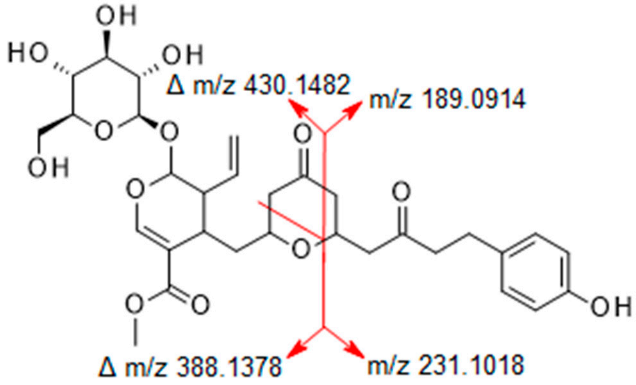

Figure E7:fragmentation hydrangenoside A (23)
