# Supplementary material for: Separation of Dihydro-Isocoumarins and Dihydro-Stilbenoids from Hydrangea macrophylla ssp. serrata by Use of Counter-Current Chromatography
Source: Molecules. 2022 May 26;27(11):3424. doi: 10.3390/molecules27113424 (PMC9182509; doi:10.3390/molecules27113424)
Supplement: Supplementary file 1 [file molecules-27-03424-s001.zip › supplementalfile_S6_flavonol.pdf]

Supplemental File S6: NMR and mass spectrometry of flavonol glycosides

Table F1: NMR assignments of flavonol glycosides

	quercetin-3-O-(alpha.1,6.rahmnopyranosyl)-sophoroside (24)			quercetin-3-O-sophoroside (25)			kaempferol-3-O-sophoroside (26)		
No	δ H (Multiplicity, *J*, nH)	δ C	HMBC	δ H (Multiplicity, *J*, nH)	δ C	HMBC	δ H (Multiplicity, *J*, nH)	δ C	HMBC
2	-	156.0	-	-	155.3	-	-	155.4	-
3	-	132.8	-	-	132.8	-	-	132.7	-
4	-	177.3	-	-	177.3	-	-	177.4	-
5	-	161.2	-	-	161.1	-	-	161.1	-
6	6.16 (d, 2.0 Hz, 1H)	98.7	93.6(8), 161.2(5), 164.3(7)	6.18 (d, 2.0 Hz, 1H)	98.6	93.3(8), 161.1(5)	6.2 (d, 2.1 Hz, 1H)	98.5	93.5(8), 161.1(5), 63.9(7)
7	-	164.3	-	-	164.2	-	-	163.9	-
8	6.36 (d, 2.0 Hz, 1H)	93.6	164.3(7)	6.39 (d, 2.0 Hz, 1H)	93.3	98.6(6), 156.1(9), 164.2(7)	6.43 (d, 2.1 Hz, 1H)	93.5	98.5(6), 156.2(9), 63.9(7)
9	-	156.4	-	-	156.1	-	-	156.2	-
10	-	103.7	-	-	103.7	-	-	103.8	-
1'	-	121.1	-	-	121.0	-	-	120.8	-
2'	7.52 (d, 2.2 Hz, 1H)	116.2	121.8(6'), 144.8(3'), 148.5(4')	7.54 (d, 2.2 Hz, 1H)	115.9	121.7(6'), 144.7(3'),155.3(2)	8.04 (m, 1H)	130.8	130.8(6'), 55.4(2), 159.8(4')
3'	-	144.8	-	-	144.7	-	6.91 (m, 1H)	115.2	115.2(5'), 20.8(1')
4'	-	148.5	-	-	148.4	-	-	159.8	-
5'	6.86 (d, 8.4 Hz, 1 H)	115.4	121.1(1'), 144.8(3'),148.5(4')	6.85 (d, 8.5 Hz, 1H)	115.3	121.0(1'), 144.7(3')	6.91 (m, 1H)	115.2	115.2(3'), 20.8(1')
6'	7.55 (dd, 8.4, 2.2 Hz, 1H)	121.8	116.2(2'), 148.5(4')	7.60 (dd, 8.5,2.2 Hz, 1H)	121.7	115.9(2'), 148.4(4')	8.04 (m, 1H)	130.8	130.8(2'), 55.4(2), 159.8(4')
A'1	5.56 (d, 7.3 Hz, 1H)	98.3	132.8(3), 76.4(A'3)	5.70 (d, 7.3 Hz, 1H)	97.8	76.7(A'3),132.8(3)	5.70 (d, 7.0 Hz, 1H)	97.8	-
A'2	3.52 (m, 1H)	82.5	-	3.49 (m, 1H)	82.7	104.1(A''1)	3.48 (m, 1H)	82.3	-
A'3	3.45 (m, 1H)	76.4	82.5(A'2)	3.13 (m, 1H)	76.7	-	3.48 (m, 1H)	76.5	-
A'4	3.14 (m, 1H)	69.5, 69.6	-	3.13 (m, 1H)	69.4	-	3.07 (m, 1H)	69.4	-
A'5	3.23 (m, 1H)	75.8	100.4(A'''1)	3.08 (m, 1H)	77.4	-	3.07 (m, 1H)	77.4	-
A'6a	3.65 (m, 1H)	66.2	-	3.49 (m, 1H)	60.5	-	3.48 (m, 1H)	60.4	-
A'6b	3.23 (m, 1H)	66.2	100.4(A'''1)	3.28 (m, 1H)	60.5	-	3.26 (m, 1H)	60.4	-
A''1	4.58 (d, 7.8 Hz, 1H)	104.1	82.5(A'2)	4.60 (d, 7.8 Hz, 1H)	104.1	74.3(A''2), 76.4(A''3),82.7(A'2)	4.61 (d, 7.8 Hz, 1H)	104	82.3(A'2)
A''2	3.07 (m, 1H)	74.3	104.1(A''1)	3.07 (m, 1H)	74.3	104.1(A''1)	3.07 (m, 1H)	74.3	-
A''3	3.14 (m, 1H)	76.5	-	3.18 (m, 1H)	76.4	-	3.17 (m, 1H)	76.4	-
A''4	3.14 (m, 1H)	69.5, 69.6	-	3.18 (m, 1H)	69.4	-	3.17 (m, 1H)	69.5	-
A''5	3.14 (m, 1H)	76.8	-	3.49 (m, 1H)	76.5	-	3.13 (m, 1H)	76.9	-
A''6a	3.56 (m, 1H)	60.7	-	3.55 (m, 1H)	60.6	-	3.59 (m, 1H)	60.7	-
A''6b	3.49 (m, 1H)	60.7	-	3.49 (m, 1H)	60.6	-	3.48 (m, 1H)	60.7	-
A'''1	4.33 (d, 1.6 Hz, 1H)	100.4	66.2(A'6b),70.3(A'''2)						
A'''2	3.34 (m, 1H)	70.3	-						
A'''3	3.23 (m, 1H)	70.5	100.4(A'''1)						
A'''4	3.05 (m, 1H)	71.8	17.7(A'''6),68.2(A'''5)						
A'''5	3.23 (m, 1H)	68.2	100.4(A'''1)						
A'''6	0.96 (d, 6.2 Hz, 3H)	17.7	68.2(A'''5),71.8(A'''4)						

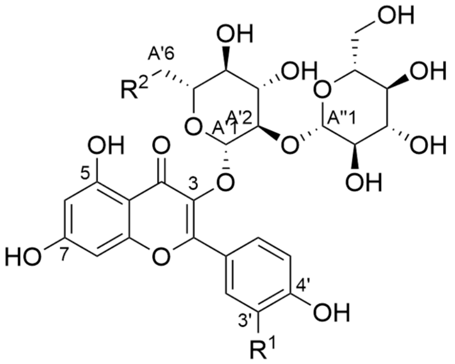

quercetin-3-O-(alpha-1,6-rahmnopyranosyl)-sophoroside (**24**) R1: OH, R2: -O-α-Rha
quercetin-3-O-sophoroside (**25**) R1: OH, R2: OH
kaempferol-3-O-sophoroside (**26**) R1: H, R2: OH

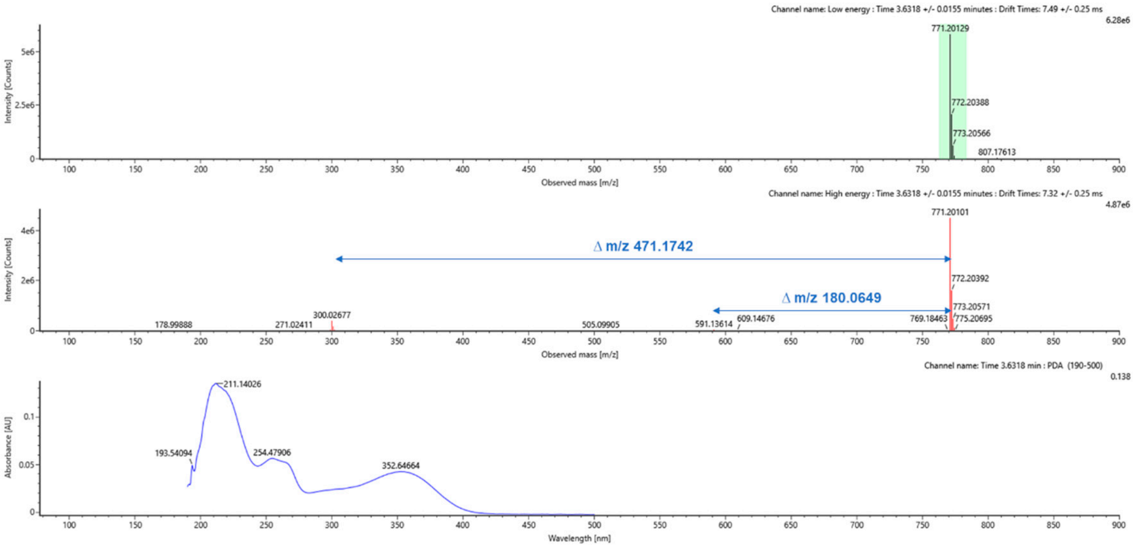

Figure F1: mass and UV-spectra of quercetin-3-O-(alpha-1,6-rahmnopyranosyl)-sophoroside (24)

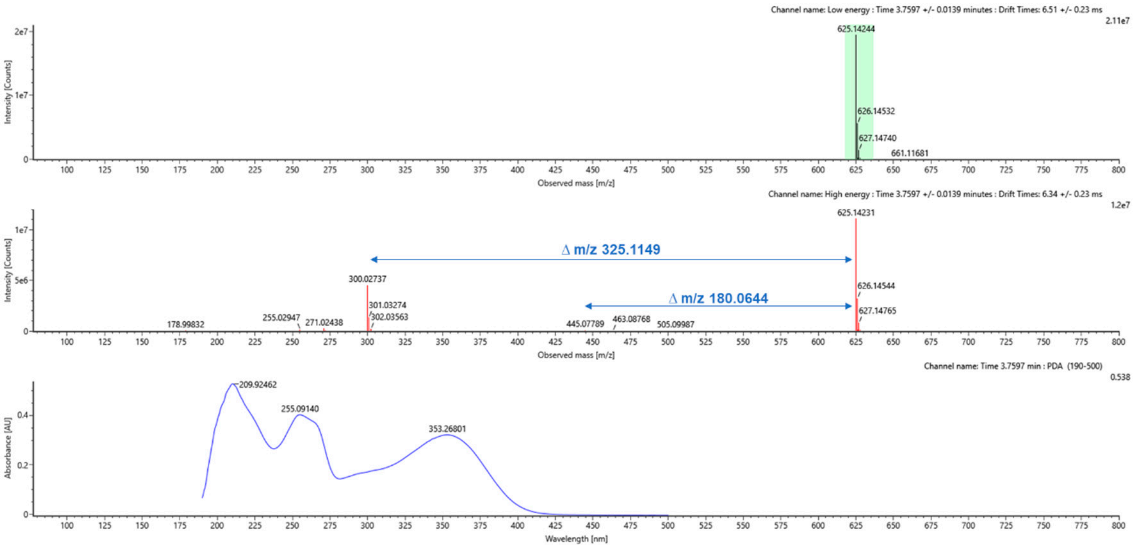

Figure F2:mass and UV-spectra of quercetin-3-O-sophoroside (25)

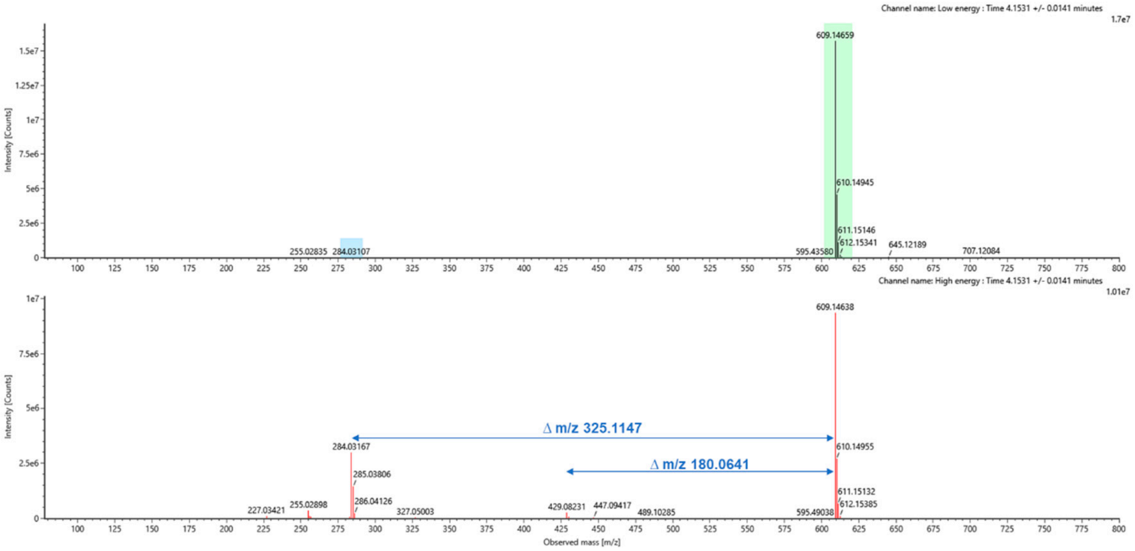

Figure F3:mass spectra of kaempferol-3-O-sophoroside (26)
