# Supplementary material for: Separation of Dihydro-Isocoumarins and Dihydro-Stilbenoids from Hydrangea macrophylla ssp. serrata by Use of Counter-Current Chromatography
Source: Molecules. 2022 May 26;27(11):3424. doi: 10.3390/molecules27113424 (PMC9182509; doi:10.3390/molecules27113424)
Supplement: Supplementary file 1 [file molecules-27-03424-s001.zip › supplementalfile_S7_compound list.pdf]

Supplemental File S7: Summarized compound list with further analytical data

Table G1: compound list with further analytical data

nr.	Compound	group	RT (min)	Precursor ion (*m/z*)	Adduct	CCS (Å²)	Characteristic mass fragments (*m/z*)
1a	hydrangenol 8-O-glucoside (isomer 1)	dihydroisocoumarin	4.37	417.1199	-H	199.54	255.0656 ($C_{15}H_{11}O_4^-$), 211.0761 ($C_{14}H_{11}O_2^-$)
1b	hydrangenol-8-O-Glc (isomer 2)	dihydroisocoumarin	4.63	417.1194	-H	201.51	255.0656 ($C_{15}H_{11}O_4^-$), 211.0761 ($C_{14}H_{11}O_2^-$)
2a	thunberginol G-3'-O-Glc (isomer 1)	dihydroisocoumarin	5.74	433.1139	-H	195.57	271.0606 ($C_{15}H_{11}O_5^-$), 227.0709 ($C_{14}H_{11}O_3^-$)
2b	thunberginol G-3'-O-Glc (isomer 2)	dihydroisocoumarin	5.82	433.1145	-H	189.44	271.0606 ($C_{15}H_{11}O_5^-$), 227.0709 ($C_{14}H_{11}O_3^-$)
3a	phyllodulcin-8-O-Glc (isomer 1)	dihydroisocoumarin	4.88	447.1296	-H	194.74	285.0767 ($C_{16}H_{13}O_5^-$), 241.0869 ($C_{15}H_{13}O_3^-$), 225.0556 ($C_{14}H_9O_3^-$)
3b	phyllodulcin-8-O-Glc (isomer 2)	dihydroisocoumarin	5.13	447.1295	-H	194.83	285.0767 ($C_{16}H_{13}O_5^-$), 241.0869 ($C_{15}H_{13}O_3^-$), 225.0556 ($C_{14}H_9O_3^-$)
4	hydrangenol-4'-O-Glc	dihydroisocoumarin	5.97	417.1187	-H	216.86	255.0658 ($C_{15}H_{11}O_4^-$), 211.0760 ($C_{14}H_{11}O_2^-$)
5	phyllodulcin-3'-O-Glc	dihydroisocoumarin	6.23	447.1298	-H	191.87	285.0763 ($C_{16}H_{13}O_5^-$), 241.0865 ($C_{15}H_{13}O_3^-$), 225.0551 ($C_{14}H_9O_3^-$)
6	thunberginol C-8-O-glucoside	dihydroisocoumarin	4.04	433.1141	-H	190.26	271.0607 ($C_{15}H_{11}O_5^-$), 227.0712 ($C_{14}H_{11}O_3^-$)
7	thunberginol E-6-O-glucoside	dihydroisocoumarin	4.95	463.1256	-H	195.82	301.0718 ($C_{16}H_{13}O_6^-$), 257.0817 ($C_{15}H_{13}O_4^-$), 241.0504 ($C_{14}H_9O_4^-$)
8	thunberginol D-6-glucoside	dihydroisocoumarin	3.78	449.1091	-H	191.28	287.0555 ($C_{15}H_{11}O_6^-$), 269.0447 ($C_{15}H_9O_5^-$), 243.0656 ($C_{14}H_{11}O_3^-$)
9	thunberginol C	dihydroisocoumarin	6.3	271.0627	-H	163.02	227.0724 ($C_{14}H_{11}O_3^-$)
10	thunberginol E	dihydroisocoumarin	6.69	301.0721	-H	173.81	283.0628 ($C_{16}H_{11}O_5^-$), 257.0834 ($C_{15}H_{13}O_4^-$), 241.0518 ($C_{14}H_9O_4^-$)
11	thunberginol G	dihydroisocoumarin	7.04	271.0610	-H	156.29	227.0711 ($C_{14}H_{11}O_3^-$)
12	hydrangenol	dihydroisocoumarin	8.15	255.0675	-H	160.24	211.0767 ($C_{14}H_{11}O_2^-$)
13	phyllodulcin	dihydroisocoumarin	8.39	285.0769	-H	171.41	225.0555 ($C_{14}H_9O_3^-$)
14	cudrabibenzyl A	dihydrostilbenic acid	3.62	436.1337	-H	196.52	273.0765 ($C_{15}H_{13}O_5^-$), 255.0658 ($C_{15}H_{11}O_4^-$), 229.0868 ($C_{14}H_{13}O_3^-$), 123.0451 ($C_7H_7O_2^-$)
15	2,4-Dihydroxy-6-[2-(-hydroxyphenyl]benzoic acid	dihydrostilbenic acid	5.84	273.0785	-H	163.00	255.0675 ($C_{15}H_{11}O_4^-$), 229.0801 ($C_{14}H_{13}O_3^-$), 123.0457 ($C_7H_7O_2^-$)
16	lunularin-4'-glucoside	dihydrostilbene	5.68	375.1454	-H	180.32	213.0919 ($C_{14}H_{13}O_2^-$), 107.0500 ($C_7H_7O^-$)
17	dihydroresveratrol-3-O-glucoside	dihydrostilbene	4.39	391.1401	-H	185.77	229.0867 ($C_{14}H_{13}O_3^-$), 123.0449 ($C_7H_7O_2^-$)
18	4-Hydroxy-8-(4-glucopyranosyl)hydroxyphenyl)octane-2,6-dione	triketide	3.15	411.1656	-H	199.00	393.1551 ($C_{20}H_{25}O_8^-$), 353.1236 ($C_{17}H_{21}O_8^-$), 231.1017 ($C_{14}H_{15}O_3^-$), 191.0706 ($C_{11}H_{11}O_3^-$)
19	taxiphyllin	cyanogenic glycoside	2.05	310.0924	-H	240.37	179.0557 ($C_6H_{11}O_6^-$)
20	deoxyloganic acid	secoiridoid	4.04	359.1345	-H	183.24	197.0814 ($C_{10}H_{13}O_4^-$), 153.0916 ($C_9H_{13}O_2^-$), 135.0812 ($C_9H_{12}O^-$)

21	loganic acid	secoiridoid	2.59	375.1297	-H	186.21	195.0653 ($C_{10}H_{11}O_4^-$), 151.0759 ($C_9H_{11}O_2^-$)
22	hydramacroside B	secoiridoid	5.59	605.2258	-H	236.88	587.2147 ($C_{30}H_{35}O_{12}^-$), 441.1409 ($C_{20}H_{25}O_{11}^-$), 413.1458 ($C_{19}H_{25}O_{10}^-$)
23	hydrangenoside A	secoiridoid	6.33	619.2397	-H	239.42	601.2286 ($C_{31}H_{37}O_{12}^-$), 231.1018 ($C_{14}H_{15}O_3^-$), 189.0914 ($C_{12}H_{13}O_2^-$)
24	quercetin-3-O-rhamnopyransyol sophoroside	flavonol	3.63	771.2013	-H	258.12	300.0268 ($C_{15}H_8O_7^-$)
25	quercetin-3-O-sophoroside	flavonol	3.76	625.1424	-H	231.49	300.0274 ($C_{15}H_8O_7^-$)
26	kaempferol-3-O-sophoroside	flavonol	4.15	609.1466	-H	231.03	284.0317 ($C_{15}H_8O_6^-$)
